# Integration of fungal transcriptomics and metabolomics provides insights into the early interaction between the ORM fungus *Tulasnella* sp. and the orchid *Serapias vomeracea* seeds

**DOI:** 10.1186/s43008-024-00165-6

**Published:** 2024-10-25

**Authors:** Silvia De Rose, Fabiano Sillo, Andrea Ghirardo, Silvia Perotto, Jörg-Peter Schnitzler, Raffaella Balestrini

**Affiliations:** 1 Institute for Sustainable Plant Protection, National Research Council, Strada Delle Cacce 73, 10135 Turin, Italy; 2 Research Unit Environmental Simulation (EUS), Helmholtz Zentrum München, Ingolstädter Landstr. 1, 85764 Neuherberg, Germany; 3 Department of Life Sciences and Systems Biology, University of Turin, Viale Mattioli 25, 10125 Turin, Italy; 4 Institute of Biosciences and Bioresources, National Research Council, Via Amendola 165/A, 70126 Bari, Italy

**Keywords:** Orchids, Symbiosis, Mycorrhizal fungi, Omics, CAZymes

## Abstract

In nature, germination of orchid seeds and early plant development rely on a symbiotic association with orchid mycorrhizal (ORM) fungi. These fungi provide the host with the necessary nutrients and facilitate the transition from embryos to protocorms. Despite recent advances in *omics* technologies, our understanding of this symbiosis remains limited, particularly during the initial stages of the interaction. To address this gap, we employed transcriptomics and metabolomics to investigate the early responses occurring in the mycorrhizal fungus *Tulasnella* sp. isolate SV6 when co-cultivated with orchid seeds of *Serapias vomeracea*. The integration of data from gene expression and metabolite profiling revealed the activation of some fungal signalling pathways before the establishment of the symbiosis. Prior to seed contact, an indole-related metabolite was produced by the fungus, and significant changes in the fungal lipid profile occurred throughout the symbiotic process. Additionally, the expression of plant cell wall-degrading enzymes (PCWDEs) was observed during the pre-symbiotic stage, as the fungus approached the seeds, along with changes in amino acid metabolism. Thus, the dual-omics approach employed in this study yielded novel insights into the symbiotic relationship between orchids and ORM fungi and suggest that the ORM fungus responds to the presence of the orchid seeds prior to contact.

**Supplementary Information:**

The online version contains supplementary material available at 10.1186/s43008-024-00165-6.

## Introduction

Like most land plants, terrestrial orchids form symbiotic relationships with soil fungi (Genre et al. 2020; Selosse et al. 2022). In their natural habitat, the symbiotic relationships between orchids and their mycorrhizal fungi play a pivotal role during the initial phases of plant growth. Although orchid seeds store lipids within the embryo, which are used in the early stage of germination (Lee et al. 2007), they generally lack sufficient internal energy resources to support germination. Therefore, for several orchid species, the symbiotic fungal partner supplies the plant with organic carbon and other essential nutrients. This trophic strategy is known as initial mycoheterotrophy (Merckx 2012). The supply of nutrients by orchid mycorrhizal (ORM) fungi influences seed germination and the subsequent development of the immature embryo into a protocorm, a postembryonic structure typical of orchids (Smith and Read 2010; Dearnaley et al. 2016). The ORM fungi are a heterogeneous group of mostly basidiomycetes belonging to different families including the families Tulasnellaceae, Ceratobasidiaceae (in the Cantharellales) and Serendipitaceae (in the Sebacinales) (Dearnaley et al. 2012). Experimental evidence from stable isotope and gene expression studies has demonstrated that, in addition to carbon (C), ORM fungi can provide the plant with phosphorus (P), nitrogen (N), and sulfur (S) (Cameron et al. 2006, 2007, 2008; Kuga et al. 2014; Fochi et al. 2017a,b; De Rose et al. 2023a). The contribution of ORM fungi to plant nutrition is observed throughout the orchid life cycle, from protocorm formation to the development of adult plants, where the fungi become mycorrhizal partners (Valadares et al. 2021). The transfer of nutrients primarily occurs across the extensive plant-fungus interface that surrounds the *pelotons*, the coiled intracellular fungal hyphae formed in ORM (Peterson and Farquhar 1994).

The advancement of *omics* technologies has provided researchers with powerful tools to investigate symbiotic mechanisms, including ORM. In the last decade, transcriptomics and metabolomics approaches have revealed intriguing molecular aspects of ORM interactions and identified potential molecular players in nutrient transfer (Perotto et al. 2014; Fochi et al. 2017a, b a,b; Ghirardo et al. 2020). In particular, RNA sequencing (RNA-seq) studies have enabled the identification of plant amino acid, oligopeptide, ammonium, and phosphate transporters, thereby providing insights into the mechanisms by which nutrients are acquired by the plant (Zhao et al. 2014; Fochi et al. 2017a,b; Miura et al. 2018; Ho et al. 2021; Kambara et al. 2023; De Rose et al. 2023b). However, while previous studies have primarily concentrated on the regulation of plant responses and transporters, there is a paucity of information on fungal genes, metabolites and associated pathways involved in the symbiotic interaction with the orchid host (Favre-Godal et al. 2020; Li et al. 2021). A proteomic approach identified the accumulation of fungal proteins involved in stress response, protein–protein interactions, and protein biosynthesis in the tissues of *Oncidium sphacelatum* during symbiosis with a *Ceratobasidium* sp. isolate (Valadares et al. 2014). Transcriptomic profiles of free-living and symbiotic mycelium of *Tulasnella calospora* inside *Serapias vomeracea* protocorms led to the identification of fungal genes coding for high and low affinity ammonium transporters and for several amino acid transporters/permeases (Fochi et al. 2017a). The expression pattern of these fungal genes, in conjunction with the expression pattern of the host plant genes, provided compelling evidence that nitrogen is transferred to the host plant as amino acids, at least in the studied species (Fochi et al. 2017a). More recently, a metabolomic approach was used to decipher the fungal response during ORM, showing the induction of structural and signalling lipids in the external fungal mycelium that developed in close proximity to the symbiotic protocorms (Ghirardo et al. 2020). These data highlighted the need for a detailed analysis of fungal gene expression remodelling and metabolomic shifts during the symbiotic relationships.

In the process of plant colonization, the first physical structure encountered by ORM fungi is the cell wall of the orchid seeds (Balestrini and Bonfante 2014; Dearnaley et al. 2016; Martin et al. 2016; Favre-Godal et al. 2020). Comparative genomic and transcriptomic studies of mycorrhizal fungi, including ORM fungi (Kohler et al. 2015), revealed that the genomes of ORM fungi belonging to the Tulasnellaceae, Ceratobasidiaceae and Serendipitaceae are characterized by an extensive set of genes coding for carbohydrate-active enzymes (CAZymes) that act on pectins, cellulose and hemicellulose. When compared with the genomes of ectomycorrhizal and endophytic fungi within Agaromycotina, the *T. calospora* genome exhibited the highest number (176) of CAZymes-related genes (Kohler et al. 2015). A significant proportion of these plant cell wall-degrading enzymes (PCWDEs) were expressed during the symbiotic stages (Kohler et al. 2015; Chen et al. 2022), thereby supporting the hypothesis that PCWDEs are functionally activated to facilitate the penetration of host cells by the fungus and thus contribute to the establishment of ORM. These enzymes undergo dynamic regulation during the interaction with the plant, as Adamo et al. (2020) found changes in the expression of ORM fungal genes encoding PCWDEs during the transition from a symbiotic to a saprotrophic interaction.

In this study, we employed both transcriptomics and non-targeted metabolomics to investigate in vitro potential changes in fungal gene expression and metabolite production during the initial stages of interactions with the host. In particular, we tested the hypotheses that (I) the ORM fungus senses and responds to the presence of *Serapias vomeracea* prior to contact with the orchid seeds; (II) the transcriptomic and metabolomic profiles of *Tulasnella* undergo significant changes during transition from pre-contact to post-contact stages; (III) the expression of fungal genes related to PCWDEs is finely tuned during the early stages of contact with orchid seeds, to favor penetration of host cell walls. To address these hypotheses, the mycelium of *Tulasnella* sp. isolate SV6 was collected at two different developmental stages prior to contact with *S. vomeracea* seeds, and as mycelium growing either in close proximity or inside symbiotic protocorms after symbiosis establishment.

## Materials and methods

### Plant and fungal materials

Seeds of *S. vomeracea* used in this study were collected during summer 2021 from mature capsules of wild plants grown in the locality of Cairo Montenotte (SV, Italy; Latitude 44.406944 N, Longitude 8.319795 E) and stored at 4 °C. The fungal isolate SV6 (MUT4178), belonging to the *Tulasnella* genus, was originally isolated from *Serapias vomeracea* roots grown in Northern Italy (Girlanda et al. 2011) and stored in the Mycotheca Universitatis Taurinensis (MUT) at the University of Turin. After thawing, vitality of the fungal isolate was verified by growing it in pure culture on solid 2% malt extract agar at 25 °C in the dark.

### Experimental design and sample preparation for transcriptomic and metabolomic analyses

**Fig. 1:**
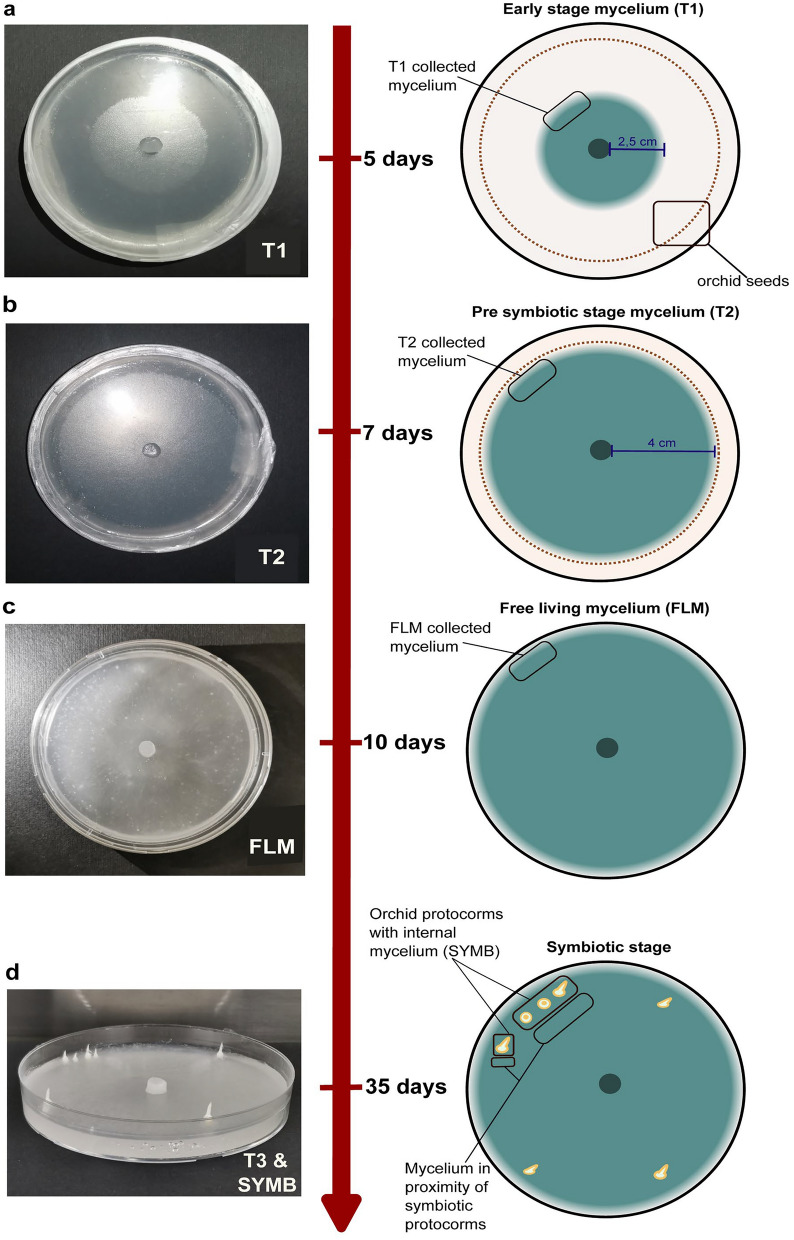
Schematic representation of the sampling design for the time course experiment. Samples coded as T1 were collected at 5 days post inoculation (d.p.i) in plates (**a**), T2 at 7 d.p.i (**b**), FLM at 10 days after plug transfer in plates (**c**), T3 and SYMB at 35 d.p.i. (**d**)

The regulation of fungal gene expression and the associated metabolomic remodelling during the initial stages of the interaction and the establishment of the symbiosis were investigated in a time-course experiment. The fungus was grown in co-culture with the plant and collected at three different time points (T1-T3) for transcriptomic (RNA-seq) and metabolomic analyses: early symbiotic stage (T1), *i.e.*, fungal mycelium before contact with the orchid seeds; pre-symbiotic stage (T2), *i.e.*, the mycelium closely reached the seeds but had no physical contact; and symbiotic stage (T3), *i.e.*, the mycelium was in the proximity of symbiotic protocorms. Additionally, we collected the symbiotic protocorms containing the fungus (namely, ‘SYMB’). A schematic representation of the sampling design is provided in Fig. 1. Co-inoculation of *Tulasnella* sp. SV6 and *S. vomeracea* seeds was performed as described in Perotto et al. (2014). Seeds were surface sterilized with a solution of 1% sodium hypochlorite and 0.1% Tween-20 for 20 min, with stirring. This was followed by three 5-min rinses with sterile distilled water. Seeds re-suspended in sterile water were then placed on dishes of autoclaved cellophane (polymeric cellulose film) membrane (Hutchinson Le Joint Français, France) previously positioned on the solid oat medium (0.3% milled oats, 1% agar). The membrane covered the surface of the 9 cm Petri dishes up to 3–4 mm from the edge, and was used to allow easy removal of the fungal material from the solid medium (Liu et al. 2010). No part of the membrane was used in the RNA extraction. A portion of actively growing mycelium was then placed in the center of each Petri dish and incubated at 20 °C in complete darkness. Samples of early-symbiotic stage (T1) were collected five days post-inoculation (dpi), when the mycelium reached approximately 5 cm in diameter, from the edge of the growing mycelium to harvest only the active growth zone; samples of the pre-symbiotic stage (T2) were collected 7 dpi (8 cm in diameter) from the edge of the growing mycelium, while samples of the symbiotic stage, consisting of mycelia grown in the proximity of (T3) and inside symbiotic protocorms (SYMB), were collected 35 dpi. Although T1 was employed as a control in the metabolomic and transcriptomic analyses, a free-living *Tulasnella* sp. SV6 mycelium (FLM) was also used for comparison. The FLM was obtained by growing the fungus on solid oat medium at 25 °C in the dark for 10 days. Samples were collected by carefully removing the mycelium (T1, T2, T3, FLM) and protocorms (SYMB) from the cellophane membrane, to ensure that no part of the membrane was used in the subsequent RNA or metabolites extraction. Three biological replicates for each condition (T1, T2, T3, SYMB, FLM) were prepared for transcriptomics and four biological replicates for metabolomics, respectively. All samples were immediately frozen in liquid nitrogen and stored at -80 °C for subsequent transcriptomic and metabolomic analyses. The SYMB sample was excluded from metabolomic analysis as it was not possible to distinguish among plant and fungal metabolites (Ghirardo et al. 2020).

### Transcriptomic analysis

For the RNA-seq experiment, protocorms (SYMB) and fungal mycelia for each tested condition (T1, T2, T3, FLM) were homogenized with TissueLyser (25 Hz, 1 min, twice, Qiagen Diagnostic GmbH, Hilden, Germany) and RNA was extracted from approximately 80 mg of biological material from each biological replicate using the “pine tree” CTAB-based method (Chang et al. 1993). The RNA, eluted in RNA-seq free water, was then quantified using NanoDrop 2000 and Qubit 4 Fluorometer (Thermo Fisher Scientific, Waltham, MA, USA). The Genartis Functional Genomics Lab (Department of Biotechnology of the University of Verona), which was responsible for library preparation and RNA sequencing, evaluated the integrity and size of the RNA-seq libraries through capillary electrophoretic analysis using the Agilent 4150 Tape Station (Agilent Technologies). The generation of the library was unsuccessful for a single biological replicate of the T2 condition (replicate 1 of T2). After poly(A) capture and following the manufacturer's instructions, 14 Illumina RNA-seq libraries were created using the TruSeq stranded mRNA ligation kit (Illumina, San Diego, CA, USA) and were quantified by real-time PCR against a standard curve with the KAPA Library Quantification Kit (KAPA Biosystems, Wilmington, MA, USA). The sequencing was performed on a Novaseq 6000 sequencer with Illumina technology in 150PE mode. The reads were aligned to the *Tulasnella calospora* AL13/4D v1.0 genome available at https://mycocosm.jgi.doe.gov/Tulca1/ (Kohler et al. 2015) using STAR v2.7.10a (Spliced Transcripts Alignment to a Reference; Dobin et al. 2013). Samtools 1.11v was used to convert SAM data into BAM files and index them (Li et al. 2009). Aligned reads on the reference genome were counted using HTseq-count 2.0.2v (Anders et al. 2014). Exon junctions and gene overlaps were mapped using the intersection method (strict).

Raw read counts were imported into the DESeq2 tool version 1.34.0 (Love et al. 2014) to identify differentially expressed genes (DEGs). Following default DESeq2 normalization of the count data (median of ratios method) and correction for multiple testing, DEG identification was carried out through Wald test and using T1 samples as controls. The three biological replicates per condition were employed to compute read count variation. To identify differentially expressed genes (DEGs), an adjusted p-value threshold of 0.05 was employed. Differentially expressed genes (DEGs) were functionally annotated in silico using Blast2GO v5.2.5 (Conesa et al. 2005), with their related Gene Ontology (GO) terms subsequently assigned. To perform a GO enrichment analysis and to provide a summary of the functions and pathways associated with the obtained sets of DEGs, Blast2GO v5.2.5 was employed. A specific database (http://pcwde.riceblast.snu.ac.kr/) was used to identify genes related to PCWDEs among the detected DEGs. Furthermore, in order to identify DEGs potentially involved in transport, a search was conducted for DEGs that included the following terms: “transporter”, “permease”, “efflux”, “carrier” and “facilitator”.

### Metabolomic analysis

A total of 16 frozen samples (four biological replicates for each condition; T1, T2, T3, and FLM) were freeze-dried at -50 °C under vacuum (Alpha 1–4 LDplus, Christ, Osterrode, Germany) and finely ground with TissueLyser (25 Hz, 1 min, Qiagen Diagnostic, Hilden, Germany). Approximately 10 mg of dried fine powder per sample was extracted following the protocol used by Bertić et al. (2021). In short, 800 µL of methanol/2-propanol/water solution (1/1/1, v/v/v) containing 50 µL of an internal standard (IS) mixture were added to the sample (Table S2). The mixture was vortexed for 1 min, sonicated in an ultrasonic bath for 10 min at 5 °C and then centrifuged at 9300 g for 10 min at 5 °C, and 400 µL of supernatant was recovered. The centrifugation process was repeated, and the remaining 400 µL of supernatant was collected to obtain a total of 800 µL of the extracted metabolite solution. The supernatant was dried by SpeedVac (Univapo 150H, Uniequip, Planegg, Germany), and re-dissolved in 350 µL of 50% (v/v) acetonitrile in water. After mixing, the solution was centrifuged at 9300 g for 10 min at 5 °C, and 300 µL of supernatant was transferred in amber glass vials for metabolomics analysis.

Non-targeted metabolomic analysis was performed as before (Bertić et al. 2021), using an ultra-performance liquid chromatography (UPLC) ultra-high resolution (UHR) tandem quadrupole/time-of-flight (QqToF) mass spectrometry (MS). The system consists of an Ultimate 3000RS UPLC (Thermo Fisher Scientific, Bremen, Germany), a Bruker Impact II (QqToF) and an Apollo II electrospray ionization source (Bruker Daltonics, Bremen, Germany). Metabolites were separated by reversed-phase liquid chromatography (RPLC) and by hydrophilic interaction liquid chromatography (HILIC), each run separately and eluted using H_2_O + 0.1% (v/v) formic acid (solvent A) and acetonitrile + 0.1% (v/v) formic acid (solvent B) (see Bertić et al. 2021 for details). The spectra were acquired in both positive ( +) and negative ( −) ionization modes. The data were processed as outlined in Bertić et al. (2021), using the software Metaboscape v4.0 (Bruker Daltonics) and the parameters given in Table S1. Results of RP and HILIC analyses were merged manually and the zero values were replaced with random numbers below a threshold value of 300 area unit. Data were normalized for the IS mixture and dry mycelium weight. Annotation of compounds by matching MS/MS spectra with the libraries ‘All Spectra’ in MoNA (Mass Bank of North America, https://mona.fiehnlab.ucdavis.edu), MS-DIAL Lipids (http://prime.psc.riken.jp), MassBank (https://massbank.eu/MassBank/), Vaniya/Fiehn Natural Products Library (available through MoNA) and GNPS (https://gnps.ucsd.edu). Mass features without MS/MS spectra were tentatively annotated on MS1 level for the precursor mass using 5.0 mDa tolerance using MetaboAnalyst 5.0 (www.metaboanalyst.ca, Pang et al. 2021). Furthermore, we used the multidimensional stoichiometric compound classification (MSCC) (Rivas-Ubach et al. 2018) approach to categorize the mass features that correspond to a molecular formula predicted by Metaboscape (‘smart formula’ tool). The MSCC algorithm confidently classifies compounds into the chemical classes ‘carbohydrates’, ‘nucleotides-related’, ‘lipids’, ‘secondary metabolites’ and ‘amino acids and protein-related’ based on their elemental ratios of C/H, C/O, C/P, and C/S (Rivas-Ubach et al. 2018).

Pathway analysis was performed using MetaboAnalyst 5.0 (Pang et al. 2021). The Mummichog 3.0 algorithm (Li et al. 2013) coupled with Gene Set Enrichment Analysis (GSEA; Subramanian et al. 2005) was employed to perform an over-representation analysis of the entire dataset of mass features, including all ( ±) ionization measurements. This analysis was performed to aid in the functional interpretation of the data. Both algorithms were operated with a p-value cutoff of 0.05 and the *Saccharomyces cerevisiae* KEGG pathway library (http://www.genome.ad.jp/kegg/). Only pathways containing at least three entries were considered.

Principal component analysis (PCA) and orthogonal partial least squares discriminant analysis (PLS-DA) were calculated using the normalized peak areas, mean centered and pareto scaled in MetaboAnalyst 5.0. PCA was employed for analyzing sample similarities by examining their associated metabolomic patterns, whereas PLS-DA for deciphering metabolomic changes between sample groups. Mass features with a score of Variable Importance of Projection (VIP) > 1 in the first component were selected and resulting mass features were blank corrected and zero filtered. Log_2_FC (using T1 as control) were calculated, applying Student t-test and Benjamini–Hochberg correction to discriminate significant values (adj. *p* value score < 0.05).

### Integration between transcriptomic and metabolomic data

The online platform Omics Fusion (https://fusion.cebitec.uni-bielefeld.de; Brink et al. 2016) was used to integrate data from RNA-seq and non-targeted metabolomics. The input data was the Log_2_FC data of DEGs (adj. *p* value < 0.05) and metabolites (VIP ≥ 0.99, FDR < 0.05, and Log_2_FC ≠ 1). To identify co-regulated transcripts and metabolites, metabolomic and transcriptomic data were used as input for optimal cluster detection and grouping via neural gas cluster analysis (Qin and Suganthan 2004) embedded in Omics Fusion, setting 2 and 5 as minimum and maximum cluster to be computed. Furthermore, a correlation analysis was conducted to investigate the relationship between metabolomic and transcriptomic data across the two stages, T2 and T3. This was achieved by calculating the Pearson correlation coefficient. A scatter plot was generated for the purpose of data visualization. The dual-stage correlation analysis enabled the identification of biological patterns that were similarly or differentially regulated across the two time points. To show the dynamic shifts in transcript abundance and metabolite contents in the different stages, alluvial plots using normalized read count and normalized mass feature peak areas, respectively, were generated. Both transcripts and metabolites were grouped based on GO terms and MSCC algorithm classification, respectively, to obtain five categories, *i.e.*, Carbohydrates, Lipids, Nucleotides, Proteins/peptides/amino acids, and Secondary metabolites.

## Results

### Distinct transcriptomic profiles of *Tulasnella* sp. SV6 during early stages of the interaction with *S. vomeracea*

**Fig. 2:**
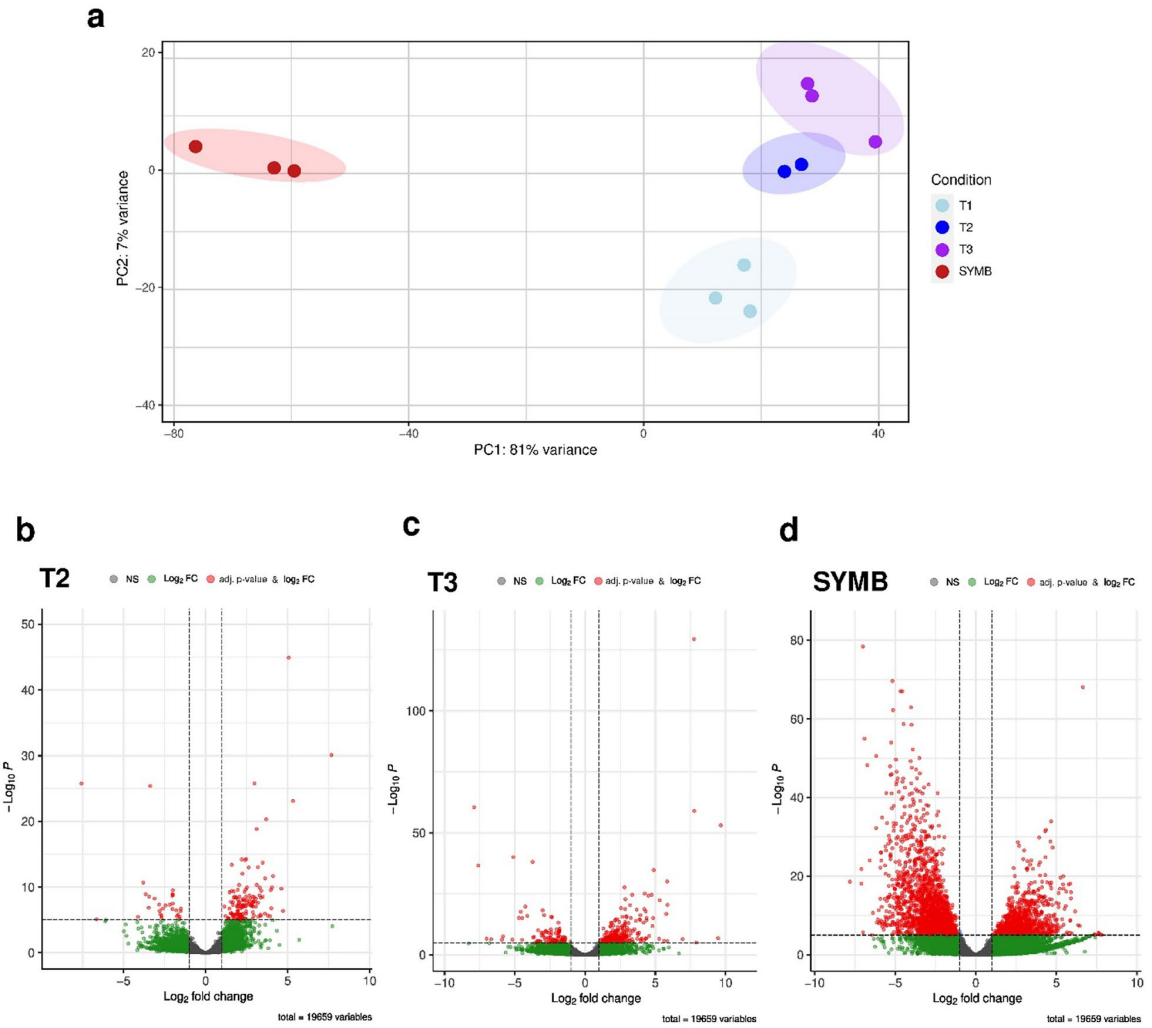
RNA-seq results. In **a**, principal components analysis (PCA) of normalized read counts of all samples used in RNAseq experiment. In **b**–**d**, Volcano plots showing identificed DEGs in T2, T3, SYMB, respectively, compared with T1 (control condition). Significant up-and down- regulated genes were represented by red dots (Log_2_FC > 1 and <  − 1, *p* adjusted value < 0.05). Green dots represented genes showing a Log_2_FC > 1 and <  − 1 and a not-significant *p* adjusted value (*p* adjusted value > 0.05). Grey dots represented genes showing both a Log_2_FC ranged from − 1 and 1, and a not-significant p-adjusted value (*p* adjusted value > 0.05)

Sequencing of RNA samples produced an average of 36 million raw reads per sample (ranging from 28,533,782 to 62,912,328; Table S3). Overall, reads mapped on the *Tulasnella calospora* AL13/4D v1.0 reference genome resulted in an average mapping rate of 72%. The mapping rate in fungus-containing protocorms (SYMB) was low (ranging from 51 to 58%) due to the presence of both fungal and plant transcripts, while samples T1, T2, T3 and FLM showed a mapping rate ranging from 75 to 80%. The total numbers of normalized read counts of *Tulasnella* sp. SV6 are reported in Table S4. The principle component analysis in Fig. 2a clearly shows that replicates of each RNA sample clustered into four distinct groups, with samples of early stage (T1), pre-symbiotic stage (T2) and mycelium grown in close proximity of symbiotic protocorms (T3) being well separate from samples of mycelium inside symbiotic protocorms (SYMB) by the first principal component (PC1), explaining 81% of the total variance. Differences between T1, T2 and T3 samples were less pronounced, as these samples were only separated in PC2, which accounted for 7% of the total variance (Fig. 2a). Fungal gene expression in T2, T3 and SYMB was compared to the early stage T1 in order to follow modulation of differentially expressed genes (DEGs) during the early events of symbiosis establishment. Sample T1 represents the earliest growth stage of the fungus in Petri dishes containing the orchids seeds, well before seed colonization, and it provided therefore a baseline condition. Additionally, gene expression in free-living mycelium (FLM) was compared with T1 samples to assess differences in gene expression between the mycelium grown in the presence (T1) and in the absence (FLM) of orchid seeds. A total of 8,153 genes (out of 19,659 *Tulasnella calospora* Al13/4D v.1 total genes) were found to be differentially regulated with adj. *p* value < 0.05. The DEGs identified in the different stages of the interactions are listed in Tables S5-S8 for T2, T3, SYMB and FLM samples, respectively. In Fig. 2b, Volcano plots showed the distribution of DEGs detected in T2, T3, SYMB, as compared to T1.

**Fig. 3:**
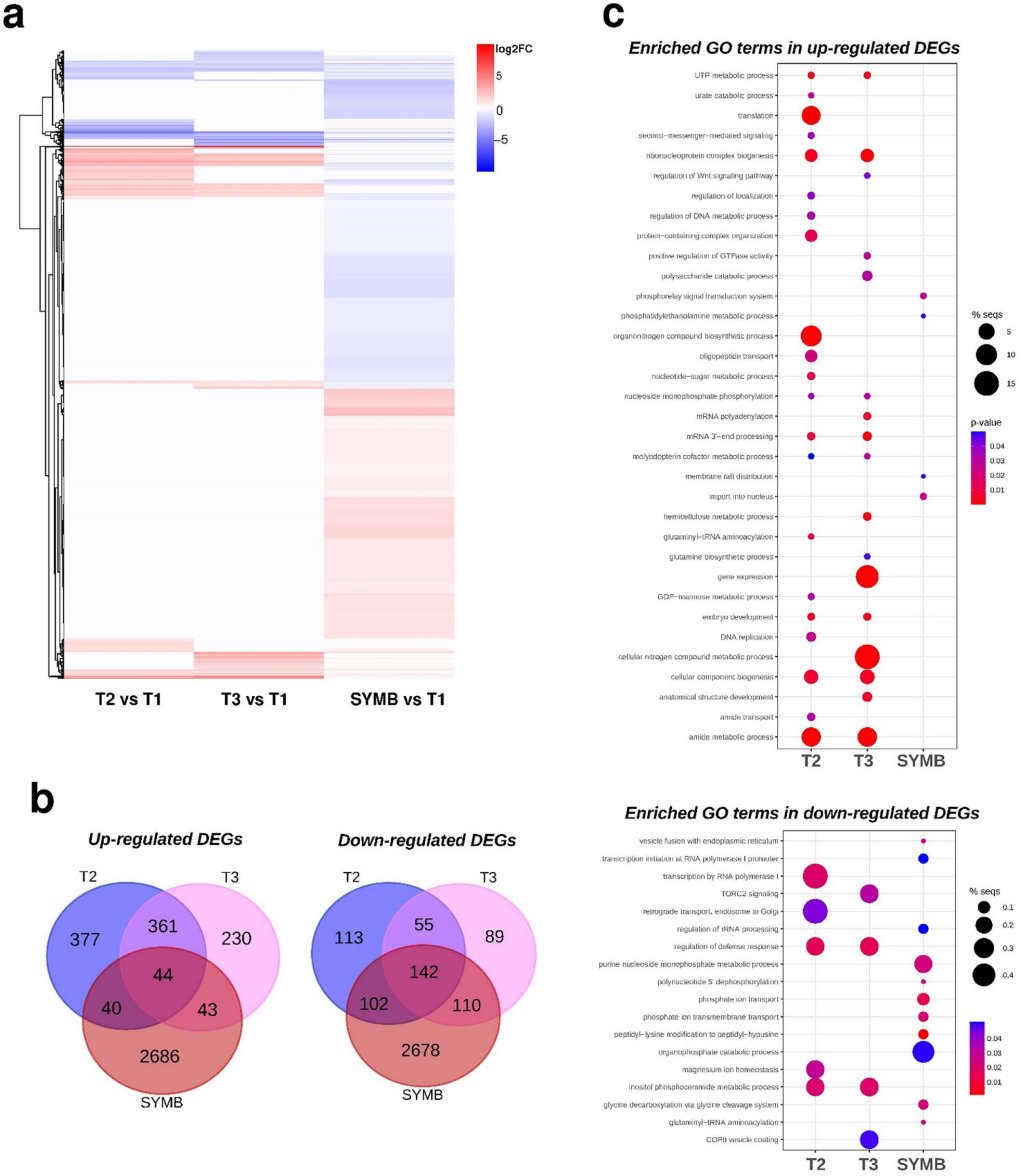
Heatmap and Venn diagrams depicting DEGs in the conditions T2, T3 and SYMB versus T1, and GO analysis results. In **a**, heatmap and hierarchical clustering of DEGs using the McQuitty algorithm. The heatmap shows the expression patterns of DEGs across three conditions compared to T1, with red indicating up-regulated and blue representing down-regulated DEGs. Different color intensity indicates different levels of expression based on Log_2_FC. In **b** Venn diagrams illustrate the overlap of up- and down-regulated DEGs, respectively, between pairwise comparisons of the conditions. In **c**, bubble plots showing GO-enriched terms classified as Biological Process (BP) in detected up and down regulated DEGs. The x-axis shows the fold enrichment values, *i.e.*, the percentage of genes in the selected DEG list belonging to a pathway divided by the corresponding percentage in the all reference gene list, and the y-axis reports the GO terms. Sizes of bubbles are proportional to the number of genes assigned to the related GO term, while bubbles color indicates the significance of the enriched term (False Discovery Rate values) as calculated by the enrichment analysis by Blast2GO

A limited transcriptomic reprogramming was observed in T2 and T3 samples in comparison to T1 (Fig. 3a). In the T2 samples, a total of 822 significantly up and 412 down-regulated fungal DEGs were found (Fig. 3b and Table S5), while T3 samples showed 678 significantly up- and 396 down-regulated DEGs (Fig. 3b and Table S6). As expected, a much more intense transcriptomic reprogramming was observed in SYMB samples (Fig. 3a). This stage showed the highest number of significantly up- and down-regulated fungal genes (2,813 up-regulated and 3,032 down-regulated DEGs; Fig. 3b and Table S7). As also expected, the FLM samples showed a very small number of DEGs when compared to the T1 samples (72 up- and 70 down-regulated genes; Table S8). A comparison of gene expression in the T2, T3 and SYMB samples revealed both shared and exclusive DEGs (Fig. 3b and Table S9). A core set of 182 genes were regulated across all conditions tested in the co-cultivation experiment. Among these DEGs, 44 transcripts increased in all three conditions and were primarily associated with fundamental cellular processes, including post-translational modifications. The other 142 DEGs were down-regulated in all three conditions and were mainly involved in protein post-translational modifications, but also in signal transduction mechanisms (Table S9). It is noteworthy that 152 DEGs (Table S9) were shared among the three conditions tested yet exhibited different regulation in the mycelium outside or inside the host plant. In particular, they were up-regulated in T2 and T3, indicating induction in the fungal mycelium growing outside the host, either prior or after contact with the plant, but down-regulated in SYMB. The annotated biological gene functions associated to these 152 DEGs were mainly related to nucleic acid metabolism, oligopeptide transport and GTPase activity. Genes respectively coding for a protein annotated as a small secreted protein (transcript ID: 242048), a carbohydrate esterase family 15 protein (transcript ID: 18233), a Cu^2^^+^ /Zn^2^^+^ superoxide dismutase SOD1 (transcript ID: 245590), and 5 proteases/peptidases (transcript IDs: 242177, 4507, 3720, 28523, 222978) were also identified as being up-regulated in T2 and T3 but down-regulated in SYMB samples (Table S9).

Some DEGs were identified exclusively in T2 (377 up-regulated and 112 down-regulated) or in T3 (230 up-regulated and 89 down-regulated) samples (Fig. 3b). In contrast, 416 DEGs were shared between T2 and T3 (Fig. 3b and Table S9) and were regulated with a similar transcriptional profile (*i.e.*, 361 up- and 55 down-regulated). Several of them encoded proteins involved in translation, ribosomal structure and biogenesis, signal transduction mechanisms, as well as genes putatively associated with transport and metabolism of lipids, amino acids, secondary metabolites, and carbohydrates (Table S9).

A total of 2686 up- and 2678 and down-regulated DEGs were found to be exclusive of the SYMB samples (Fig. 3b). This large DEGs dataset featured genes associated with transmembrane monodehydroascorbate reductase activity, intracellular trafficking, secretion and vesicular transport, cell wall/membrane/envelope biogenesis, transport and metabolism of macromolecules. Among the DEGs exclusive of SYMB samples, most genes putatively encoding glutathione S-transferases (9 out of 10) were down-regulated, whereas a glutathione synthase (transcript ID: 70710) was up-regulated (Table S9).

### GO enrichment analysis suggests dynamic changes in metabolic processes before and after contact with the host

A GO enrichment analysis was conducted to identify the biological processes, molecular functions and signalling pathways that were involved in the early interactions between the fungus and the orchid seeds. The DEGs were grouped into functional classes, based on their putative involvement in specific biological processes (Fig. 3c). The GO terms in the T2 up-regulated transcriptome were mainly related to translation, organonitrogen compound biosynthetic process, amide metabolic process and oligopeptide transport (Fig. 3c). In the T3 transcriptome, the most prevalent GO terms among the up-regulated genes were those involved in the cellular nitrogen compound metabolic process and gene expression (Fig. 3c). Among the down-regulated genes, the main enriched GO terms in T2 and T3 were involved in the regulation of defense response and inositol phosphoceramide metabolic process (Fig. 3c). Four different significant GO terms were identified for the up-regulated genes in the SYMB condition, although with a low percentage of sequences, and were related to the phosphorelay signal transduction system, import into nucleus, membrane raft distribution and phosphatidylethanolamine metabolic process (Fig. 3c). The GO terms enriched for the down-regulated genes in SYMB were mainly related to the organophosphate catabolic process (Fig. 3c).

### Some fungal genes are up-regulated exclusively in the pre-symbiotic stage of the interaction (T2)

To investigate the influence of orchid seeds on the fungal transcriptome prior to contact with the plant, we focused on the top 20 DEGs up-regulated exclusively in the pre-symbiotic stage T2 (Table 1). The analysis revealed genes putatively encoding a PIH1/Nop17-domain-containing protein, a heat shock protein (HSP) family 20, a calmodulin, an oligopeptide transporter, an amphiphysin Hob1p, and a priA protein suggested to be involved in fungal fruiting body development (Yan and Ma 2024). A chitin synthase belonging to the Glycosyl Transferase (GH) Family 2 was also identified among the 20 most highly up-regulated genes in T2, in conjunction with an amino acid/polyamine transporter I, an ABC transporter, and two vegetative incompatibility proteins. In addition to the aforementioned top 20 up-regulated genes, other relevant genes within the entire dataset of T2 up-regulated DEGs (Table S5) included those coding for an additional HSP20-like chaperone (transcript ID: 241977, Log_2_FC: 1.13), a myosin (transcript ID: 242888, Log_2_FC: 1.55), and a calmodulin (transcript ID: 241437, Log_2_FC: 1.68). Furthermore, putative kinase-like proteins, which are also involved in signal transduction mechanisms (Kosti et al. 2010), exhibited a slight increase in expression, as well as a C-type lectin (transcript ID: 17225, Log_2_FC: 1.05). In the amino acid metabolism, two dehydrogenases were up-regulated, one NADP-specific for glutamate (transcript ID: 77118, Log_2_FC: 2.07) and one homoserine specific (transcript ID: 79986, Log_2_FC: 1.18). Additionally, genes coding for asparagine synthase (transcript ID: 66686, Log_2_FC: 1.2), and a cobalamin-independent methionine synthase (transcript ID: 96062, Log_2_FC: 1.49) were identified.

**Table 1:** List of top 20 DEGs up-regulated exclusively in T2 stage, as compared to T1

Transcript ID	Log_2_FC	Log_2_FCSE	Adj. *p* value	Blastx (best identified match) [species]	Accession	*e*-value	Identity (%)
19022	3.61	0.63	1.15E−06	Pre-RNA processing PIH1/Nop17-domain-containing protein [Pisolithus orientalis]	XP_051595886.1	1e−49	38.83
66522	3.43	1.16	3.73E−02	HSP20-like chaperone [Suillus paluster]	XP_041178010.1	2e−18	46.39
18523	3.35	0.60	2.56E−06	calmodulin [Rhodofomes roseus]	XP_047772407.1	6e−27	40.00
75873	3.29	0.77	7.46E−04	OPT oligopeptide transporter protein [Rhizoctonia solani]	XP_043179825.1	2e−67	76.06
246137	3.26	0.67	7.06E−05	Protein hob1 [Psilocybe cubensis]	XP_047754336.1	9e−109	62.11
27465	2.87	0.87	1.65E−02	Protein priA [Psilocybe cubensis]	XP_047743471.1	8e−12	30.14
58159	2.76	0.71	2.95E−03	Vegetative incompatibility protein [Colletotrichum fructicola]	XP_031875660.1	9e−10	33.33
67015	2.72	0.82	1.59E−02	SSU ribosomal protein S9P [Lentinula edodes]	XP_046085726.1	3e−48	70.37
247088	2.69	0.57	1.06E−04	40S ribosomal protein S17 [Lentinula edodes]	XP_046091369.1	4e−64	86.61
75118	2.66	0.55	8.57E−05	Amino acid/polyamine transporter I [Filobasidium floriforme]	XP_046040051.1	6e−70	59.47
22519	2.49	0.81	2.87E−02	Urate oxidase [Kwoniella mangroviensis CBS 8507]	XP_019001393.1	1e−39	66.67
79704	2.47	0.59	9.81E−04	Glycosyltransferase family 2 protein [Suillus discolor]	XP_041298901.1	0.0	68.27
22722	2.31	0.70	1.54E−02	Kinase-like domain-containing protein [Suillus subalutaceus]	XP_041251532.1	1e−04	32.06
74495	2.29	0.72	2.16E−02	60S ribosomal protein L30-1 [Psilocybe cubensis]	XP_047747268.1	1e−32	81.97
55002	2.25	0.74	3.09E−02	Sacsin [Psilocybe cubensis]	XP_047751045.1	2e−16	58.46
78780	2.25	0.74	3.15E−02	HAD-like protein [Aureobasidium namibiae CBS 147.97]	XP_013429611.1	2e−85	52.26
35692	2.14	0.64	1.45E−02	putative phosphatase HAD1 [Psilocybe cubensis]	XP_047750376.1	8e−30	59.76
19766	2.13	0.47	2.81E−04	ABC transporter [Rhizoctonia solani]	XP_043186107.1	8e−106	50.14
245006	2.12	0.51	1.26E−03	40S ribosomal protein S13 [Laccaria bicolor S238N-H82]	XP_001885801.1	2e−85	79.47
4711	2.11	0.67	2.33E−02	aldo/keto reductase [Cryphonectria parasitica EP155]	XP_040772933.1	4e−26	50.49

### Genes exclusively up-regulated during symbiotic stages (T3 and SYMB) include several hydrolases as well as genes involved in primary metabolism

Specific metabolic changes have been previously identified in the mycelium growing in the close proximity of mycorrhizal orchid protocorms (T3; Ghirardo et al. 2020). However, gene expression in this stage of the symbiosis has never been investigated. Here, we compared gene expression in the external mycelium at stage T3 with the earliest symbiotic stage (T1), and focused on the top 20 DEGs exclusively up-regulated in T3 (Table 2; Fig. 1). Genes putatively coding for two α/β hydrolase proteins, an aminopeptidase puromycin sensitive, a serine/threonine-protein phosphatase ppe1, and a quinone oxidoreductase 15 were identified. Additionally, genes coding for a zinc metalloprotease, a Zn-dependent exopeptidase, a DsbA protein belonging to the Dsb (disulfide bond) enzymes family and three Carbohydrate Active enzymes (CAZymes) genes coding for Glycoside Hydrolases, belonging to Family 10, 11 and 16 were also strongly up-regulated in T3. Other relevant up-regulated genes (Table S6) code for proteins involved in lipid metabolism, including a sphingolipid C9-methyltransferase (transcript ID: 85119, Log_2_FC: 1.59) and a protein containing a beta subunit of fatty acid synthase (transcript ID: 216966, Log_2_FC: 2.29). Together with proteins involved in sulfate assimilation and reduction such as phosphoadenosine phosphosulfate reductase (PAPS reductase, transcript ID: 241376, Log_2_FC: 1.69), this gene is also up-regulated.

**Table 2:** List of top 20 DEGs up-regulated exclusively in T3 stage, as compared to T1

Transcript ID	Log_2_FC	Log_2_FCSE	Adj. *p* value	Blastx (best identified match) [species]	Accession	*e*-value	Identity (%)
57403	5.86	1.08	2.90E−06	Epoxide hydrolase [Rhizoctonia solani]	XP_043182524.1	2e−131	51.54
34573	4.83	1.25	2.20E−03	Peptidase family M1 protein [Rhizoctonia solani]	XP_043187531.1	3e−26	72.06
21101	4.64	1.08	4.21E−04	Alkylated DNA repair protein alkB 6 [Malassezia restricta]	XP_027483908.1	5e−57	42.28
17709	4.58	1.02	1.87E−04	F-box-like domain-containing protein [Rhizoctonia solani]	XP_043180427.1	8e−09	33.67
241080	4.36	0.51	7.85E−15	Serine/threonine-protein phosphatase ppe1 [Sparassis crispa]	XP_027618106.1	0.0	79.50
23474	4.04	0.68	2.09E−07	Glycosyl hydrolases family 11-domain-containing protein [Lasiosphaeria miniovina]	XP_060296216.1	6e−49	64.39
242728	3.97	0.97	9.21E−04	Quinone oxidoreductase 15 [Heterobasidion irregulare TC 32-1]	XP_009552503.1	2e−50	40.18
18269	3.95	0.72	2.15E−06	TPR-like protein [Punctularia strigosozonata HHB-11173 SS5]	XP_007389065.1	2e−90	37.20
16959	3.62	0.88	8.38E−04	Fungal-specific transcription factor domain-containing protein [Suillus subalutaceus]	XP_041249040.1	2e−22	70.37
226035	3.43	0.65	6.11E−06	Extracellular metalloprotease [Rhizoctonia solani]	XP_043176449.1	3e−131	65.96
24416	3.35	0.68	3.54E−05	Kinase-like domain-containing protein [Cantharellus anzutake]	XP_038918764.1	7e−32	30.72
24036	3.35	0.63	5.86E−06	Cytochrome P450 family protein [Rhizoctonia solani]	XP_043185935.1	3e−112	43.02
31503	3.27	0.71	1.19E−04	Glycoside hydrolase family 16 protein [Laccaria bicolor S238N-H82]	XP_001887475.1	6e−103	53.06
240495	3.09	0.79	1.73E−03	DSBA-like thioredoxin domain protein [Rhizoctonia solani]	XP_043179560.1	2e−48	44.88
28826	3.06	0.70	3.07E−04	Alpha/Betahydrolase YheT [Dacryopinax primogenitus]	XP_040623534.1	7e−07	26.81
225980	3.04	0.73	8.15E−04	Sodium/inorganic phosphate symporter [Rhizoctonia solani]	XP_043186227.1	0.0	55.29
231976	3.02	0.72	6.95E−04	Endo-beta-1,4-xylanase [Rhizoctonia solani]	XP_043180145.1	8e−111	68.01
22670	2.98	0.66	1.73E−04	Zn-dependent exopeptidase [Gloeophyllum trabeum ATCC 11539]	XP_007863390.1	4e−87	43.27
215906	2.97	0.70	5.00E−04	ATP-dependent RNA helicase DBP7 [Psilocybe cubensis]	XP_047752736.1	0.0	54.50
163733	2.91	0.40	9.24E−11	Glycerophosphocholine permease GIT4 [Sparassis crispa]	XP_027618949.1	9e−167	59.10

The top 20 DEGs up-regulated exclusively in the SYMB samples (Table 3) included genes coding for CAZymes belonging to Glycoside Hydrolase Family 30, 43 and 79, two CAZymes belonging to Polysaccharide Lyase Family 1, and a putative cellulose hydrolase harboring a Carbohydrate-Binding Module Family 1. In addition, other strongly up-regulated genes included an aquaporin-like, a putative DNA helicase ATP-dependent, a reductase belonging to SDR family and a galactose oxidase. Two putative kinase-like domain-containing proteins as well as a putative serine/threonine protein kinase and a dsk1 protein kinase were also detected (Table 3), and a putative GTPase Rab6 belonging to small G protein superfamily (transcript ID: 35046, Log_2_FC: 6.09). Furthermore, several transcripts putatively involved in amino acid metabolism were identified among the DEGs up-regulated exclusively in SYMB condition (Table S9), from biosynthesis (e.g., acetolactate synthase—transcript IDs 21435, Log_2_FC: 2.23 -, prephenate dehydratase—12078, Log_2_FC: 1.82 -, anthranilate synthase—71095, Log_2_FC: 1.60 -, argininosuccinate lyase—245918, Log_2_FC:1.91), to catabolism (e.g., ornithine cyclodeaminase – 8359, Log_2_FC: 1.33 -, ornithine carbamoyltransferase—224256, Log_2_FC: 1.92-, lysine-ketoglutarate reductase—215804, Log_2_FC: 1.56). Genes coding for a putative PAPS reductase, which is involved in the sulfate reduction pathway (242229), and two putative enzymes involved in the metabolism of sulfur-containing amino acids (*i.e.*, a cystathionine gamma-synthase—214720 and a cysteine desulfurase NFS1—17165) were also found to be up-regulated (Log_2_FC: 1.77, 2.80 and 4.63, respectively).

**Table 3:** List of top 20 DEGs up-regulated exclusively in SYMB stage

Transcript ID	Log_2_FC	Log_2_FCSE	Adj. *p* value	Blastx (best identified match) [species]	Accession	*e*-value	Identity (%)
43357	7.81	1.61	8.61E−06	Uncharacterized protein LACBIDRAFT_395470 [Laccaria bicolor S238N-H82]	XP_001888293.1	3e−43	47.25
26909	7.62	1.50	2.91E−06	Glycoside hydrolase family 30 protein [Trichoderma asperellum CBS 433.97]	XP_024760871.1	3e−84	74.58
53692	7.04	1.51	2.05E−05	Serine/threonine/tyrosine-protein kinase HT1 [Psilocybe cubensis]	XP_038918764.1	1e−28	31.70
247044	6.82	1.52	4.41E−05	glycoside hydrolase family 43 protein [Macroventuria anomochaeta]	XP_033558161.1	2e−42	59.42
35773	6.82	1.55	6.62E−05	ATP-dependent DNA helicase [Mycena indigotica]	XP_037223538.1	3e−32	42.62
27138	6.73	1.53	6.12E−05	cellulose hydrolase, putative [Aspergillus clavatus NRRL 1]	XP_001275546.1	1e−06	66.67
242138	6.64	1.56	0.000314	aquaporin-like protein [Dacryopinax primogenitus]	XP_040631163.1	3e−71	58.06
31239	6.24	1.55	0.000395	Pectate lyase [Rhizoctonia solani]	XP_043184226.1	8e−23	55.06
35046	6.09	1.52	0.000303	Ras-domain-containing protein [Coniophora puteana RWD-64-598 SS2]	XP_007772255.1	4e−35	35.53
32454	6.09	1.56	0.000642	Polyubiquitin [Schizophyllum commune H4-8]	XP_003030030.1	2e−54	42.40
247151	5.95	0.71	4.33E−15	Oxidoreductase [Gloeophyllum trabeum ATCC 11539]	XP_007864658.1	9e−61	44.67
246267	5.89	0.91	2.14E−09	Glycoside hydrolase family 79 protein [Desarmillaria tabescens]	XP_060333592.1	3e−15	48.08
25533	5.85	1.57	0.000843	Putative TIM8-translocase of the mitochondrial inner membrane [Jaminaea rosea]	XP_025361616.1	9e−15	55.56
28722	5.85	1.61	0.001277	Galactose oxidase [Stereum hirsutum FP-91666 SS1]	XP_007306347.1	3e−65	74.44
226373	5.81	1.44	0.000307	Kinase-like protein [Fomitiporia mediterranea MF3/22]	XP_007265203.1	2e−36	40.96
25837	5.76	1.58	0.001128	Pectin lyase-like protein [Stereum hirsutum FP-91666 SS1]	XP_007306015.1	4e−34	50.38
12135	5.74	1.54	0.000867	Nuclear protein 96-domain-containing protein [Suillus clintonianus]	XP_041208265.1	3e−40	38.16
84363	5.74	1.10	1.98E−06	Putative E3 ubiquitin-protein ligase rbrA [Colletotrichum siamense]	XP_036502071.1	2e−09	49.15
246249	5.67	1.58	0.001408	Phosphatidylserine decarboxylase [Kwoniella mangroviensis CBS 8507]	XP_019005511.1	2e−17	53.85
51640	5.64	1.47	0.000577	Kinase dsk1 [Fusarium graminearum PH-1]	XP_011323018.1	3e−14	44.30

### Gene expression possibly involved in fungal responses to external symbiotic signals

To identify fungal pathways potentially involved in the early response to plant signals (currently unknown in ORM), we selected those DEGs coding for fungal proteins putatively involved in signal transduction mechanisms, according to their KOG or description (Table S9). Differences in gene expression between FLM and T1 were minimal, indicating that the fungus at the initial stage of co-culture (T1) did not perceive (or respond to) the presence of the orchid seeds. By contrast, genes belonging to the KOG group “Signal transduction mechanisms” were up-regulated in later stages of co-culture (Table S9). A limited number of DEGs were shared by all different growth stages in co-culture, the majority of them being down-regulated in all samples (T2, T3 and SYMB). One exception was a gene coding for a divergent CRAL/TRIO domain-containing protein (223214), that was up-regulated in all three conditions (T2, T3 and SYMB). The CRAL/TRIO motif is a common feature to several proteins that bind small lipophilic molecules (Panagabko et al. 2003; Gupta et al. 2012).

All DEGs shared by the external mycelium, either prior (T2) or after (T3) symbiosis establishment, showed the same regulation (i.e., up- or down-regulation), indicating a fungal response irrespective of the expression pattern inside the symbiotic protocorms (Table S9). Genes up-regulated in the T2 mycelium are of particular interest, as they suggest a fungal response to plant signals prior to contact. Among the genes exclusively up-regulated in T2 were two genes coding for calmodulin (transcript IDs: 18523 and 241437), with an average expression levels of Log_2_FC ± SD: 2.5 ± 1.1 (Table 4). A single calmodulin gene was identified as DEG in T3 and SYMB conditions, but with lower average expression levels (Table 4). Two genes coding for kinase-like domain-containing proteins (transcript IDs: 66501 and 72438, respectively) were exclusively up-regulated in T2, as well as a gene coding for a myosin regulatory light chain cdc4 (transcript ID: 242888), a gene coding for a small G protein (transcript ID: 30953) and two genes coding for ran-specific GTPase-activating proteins (transcript IDs: 12828 and 242041).

**Table 4:** Main gene categories putatively involved in signalling processes in the different tested conditions

Category		T2	T3	SYMB	FLM
Kinase-like proteins	Number of DEGs	47	39	266	6
	average expression (log_2_FC ± SD)	−0.109 ± 1.938	−0.179 ± 2.63	0.854 ± 1.498	−0.641 ± 2.42
Calmodulin-like proteins	number of DEGs	2	1	1	0
	average expression (log_2_FC ± SD)	2.515 ± 1.181	2.591 ± 0.0	1.937 ± 0.0	NA
GTPase-like proteins	number of DEGs	4	6	29	2
	average expression (log_2_FC ± SD)	−0.223 ± 2.060	1.177 ± 2.493	1.143 ± 2.314	−0.514 ± 2.907
C-type lectins	number of DEGs	1	0	4	0
	average expression (log_2_FC ± SD)	1.05 ± 0.0	NA	−1.144 ± 1.612	NA

Number of DEGs detected, when compared to control condition (T1), and “function” expression values (as the expression average value of all the genes putatively annotated within the same category) are reported

DEGs putatively coding for kinase-like proteins were found both in T2 and T3 samples (47 and 39 in T2 and T3, respectively), as well as GTPase-like proteins (4 and 6 in T2 and T3, respectively; Table 4). In particular, the most up-regulated gene in T2 was identified as a putative Ras guanine-nucleotide exchange factor, or RasGEF (transcript ID: 27558, Log_2_FC: 3.69). This gene plays a role in the signalling cascade involving Ras and Ras‐like proteins (Chen et al. 2020). The same gene was also found to be up-regulated in T3, although at a lesser extent (Log_2_FC: 2.96), whereas it was not regulated in SYMB. Another RasGEF (transcript ID: 242442) was up-regulated in both T2 and T3, but strongly down-regulated in SYMB samples. Another gene which may be involved in the downstream RAS signalling pathway (Mizunuma et al. 2013) was identified as a regulatory subunit of the cAMP-dependent protein kinase (transcript ID: 9609, Log_2_FC: 1.25), and was specifically up-regulated in T2.

The largest number of regulated genes with a potential role in signal transduction mechanisms were identified in SYMB, with 20 of them exhibiting a Log_2_FC expression level greater than 4 (Table 4). The SYMB samples showed a considerable number of DEGs related to kinase-like proteins (a total of 266 up- and down-regulated DEGs) and GTPase-like proteins (a total of 29 up and down regulated DEGs). The average expression level of kinase-like proteins in the SYMB condition was Log_2_FC ± SD: 0.8 ± 1.5 (Table 4).

### Expression of CAZymes and PCWDEs in *Tulasnella* sp*.* SV6 in co-culture with *S. vomeracea* suggests their involvement in symbiosis establishment

**Fig. 4:**
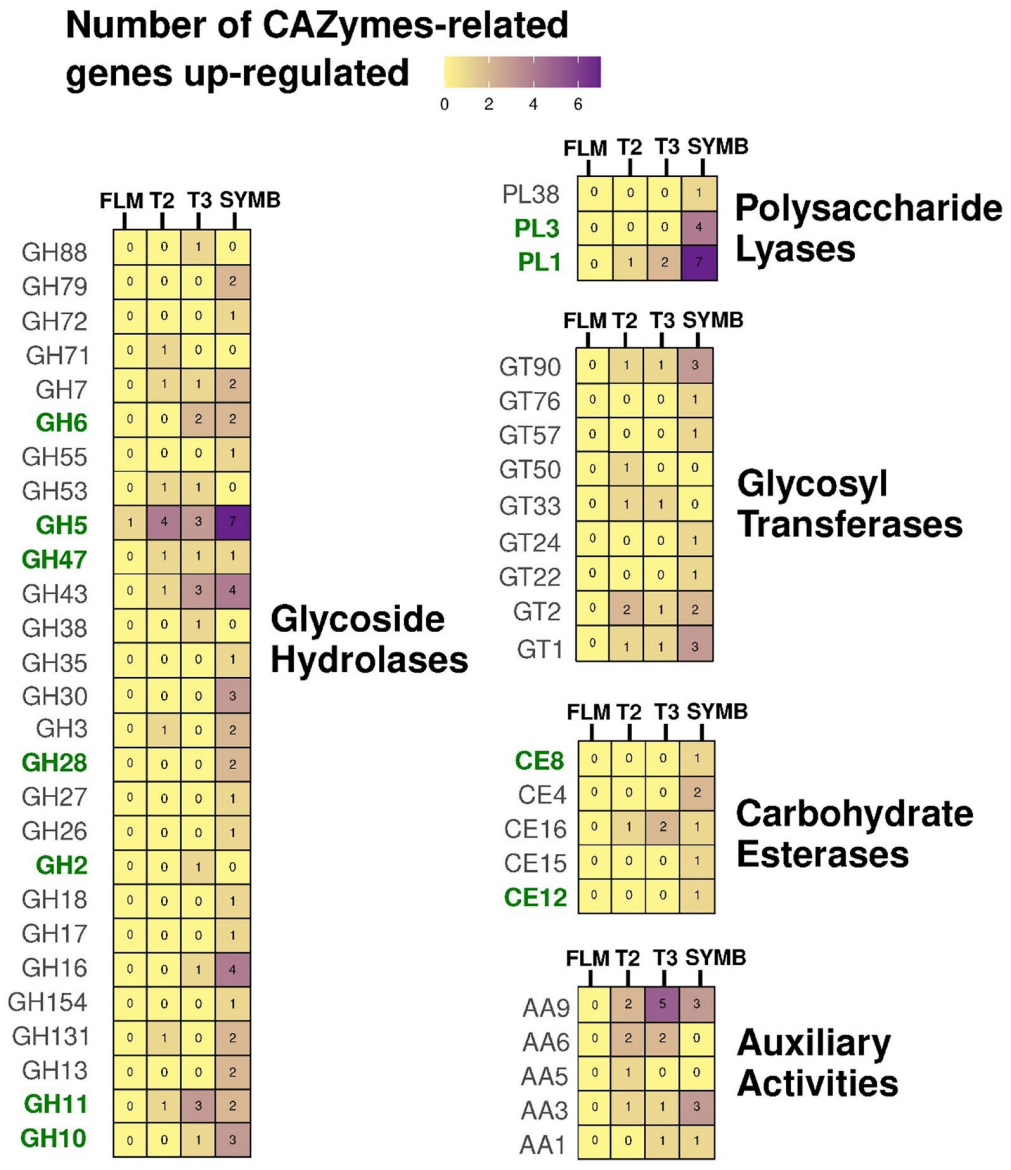
Number of CAZymes-related genes up-regulated in the three conditions. Color intensity is related to number of up-regulated genes corresponding to the five CAZYmes categories identified. Enzymes corresponding to PCWDEs are in bold and green colored

The expression of fungal genes putatively coding for CAZymes was found to be subject to different regulatory mechanisms during the different stages of the symbiosis establishment. In T2, 25 genes associated with CAZymes were identified as being up-regulated (Fig. 4), in addition to a Glycosyl Transferase Family 2-related gene that was among the top 20 most up-regulated genes (Table 1). In T3 and SYMB, the number of up-regulated CAZymes-related genes increased to 36 and 83, respectively (Fig. 4). Of the 176 CAZymes-related genes annotated in the *T. calospora* AL-13/4D v1.0 genome, 100 were classified as coding for PCWDEs based on the PCWDE database. Among the up-regulated genes in the three conditions tested, those belonging to the Glycoside Hydrolase (GH), Polysaccharide Lyase (PL), and Carbohydrate Esterase (CE) families of enzymes were identified as PCWDEs. In particular, the gene set included GH5 and former GH61 (now AA9), which are known to act on cellulose; GH10 and GH11, which are known to act on xylans (Puchart et al. 2021; Gavande et al. 2023); GH47 and GH2, which mainly target α and β-mannosides and β-D-galactosides (Herscovics 2001; Talens-Perales et al. 2016); GH28, PL4, PL3, and PL1, which target pectins (Bonnin and Pelloux 2020); and CE8 and CE12, which act on hemicelluloses and xylans through deacetylation (Gavande et al. 2023); GH28, PL4, PL3, and PL1, which target pectins (Bonnin and Pelloux 2020); CE8 and CE12, which act on hemicelluloses and xylans through deacetylation (Gavande et al. 2023). Seven of the genes (four belonging to the GH5 family, a GH47, a GH11 and a PL1) were found to be upregulated in T2, 13 (three belonging to the GH2 family, two belonging to the GH6 family, a GH47, three belonging to the GH11 family, a PL1 and two belonging to the PL2 family) in T3, and 30 in SYMB (Fig. 4). In contrast, eight PCWDE-related genes were only detected in the SYMB condition. It is noteworthy that certain genes encoding CAZymes in families GH5 (transcript IDs: 67006 and 20384), GH7 (transcript ID: 24394) and AA9 (transcript IDs: 218573 and 6298) showed increased expression in T2 and T3 yet exhibited decreased or no significant regulation in SYMB samples (Table S9). A single DEG corresponding to a GH5 was identified in the FLM when compared to T1 (Fig. 4).

### Expression of membrane transporters in *Tulasnella* sp. SV6 in co-culture with *S. vomeracea*

Several DEGs were putatively identified as transporters. None of the transporters identified as DEGs in T2 and T3 were found to be regulated in the FLM condition, thereby confirming a similar gene expression during saprotrophic growth (FLM) and the early T1 stage. This also suggests that DEGs encoding transporters in T2 and T3 were somehow related to the presence of the host rather than by exploitation of the substrate. A total of 198 DEGs encoding putative transporters were found in our dataset (Table S10). Of the 19 DEGs putatively involved in transport exclusively detected in T2, all were up-regulated except for one gene associated with copper movement, which was down-regulated (transcript ID: 245837, Log_2_FC: -2.29). In addition to the strongly up-regulated transporters already mentioned, different genes coding for major facilitator superfamily (MFS) proteins were identified, together with a putative urea transporter (transcript ID: 243251, Log_2_FC: 1.57), an inorganic phosphate permease (transcript ID: 15435, Log_2_FC: 1.42), three ABC transporters, one of which belongs to the Pleiotropic Drug Resistance (PDR) proteins (transcript ID: 19766, Log_2_FC: 2.13) and one protein possibly involved in iron export (transcript ID: 240789, Log_2_FC: 1.22). Exclusively up-regulated in T2 were also a member of Yellow stripe like 5 (YSL5) metal-nicotianamine transporter (transcript ID: 228210, Log_2_FC: 1.97) and two thiamine transporters thi9 (transcript IDs: 33469, Log_2_FC: 1.73 and 29197, Log_2_FC: 1.43).

Only six genes encoding transporters were differentially expressed exclusively in T3. Among them, a putative UDP-xylose and UDP-N-acetylglucosamine transporter (transcript ID: 33298, Log_2_FC: -3.47) was strongly down-regulated, while a phospholipid transporter (transcript ID: 163733) showed up-regulation (see Table 2).

As expected, a considerably greater number of DEGs encoding putative transporters was identified in SYMB. Among the 142 up- and down-regulated fungal transporters exclusively found in symbiotic protocorms, several were general substrate transporters belonging to the major facilitator superfamily (MFS) and the ATP-binding cassette (ABC) superfamilies, as well as different amino acid and oligopeptide transporters/permeases (Table S9). Highly up-regulated genes related to lipid and carbohydrate transport in SYMB included a phospholipid transporter (transcript ID: 211343; Log_2_FC: 3.17) and a protein containing a triose-phosphate transporter family domain (transcript ID: 247150; Log_2_FC: 4.41). In addition, genes coding for a phospholipid-translocating P-type ATPase (transcript ID: 25698) and a GDP-mannose 1 (transcript ID: 69923) transporter (Log_2_FC: 1.65 and 1.30, respectively) were identified. Only an UDP-galactose transporter (transcript ID: 160960), was identified in the FLM. This transporter was shared with the symbiotic condition and was down-regulated in both the SYMB and FLM stages, with respective Log_2_FC values of -2.40 and -1.43.

### Metabolomic profiling of pre-symbiotic and symbiotic mycelial growth highlights changes mainly in lipid metabolism

**Fig. 5:**
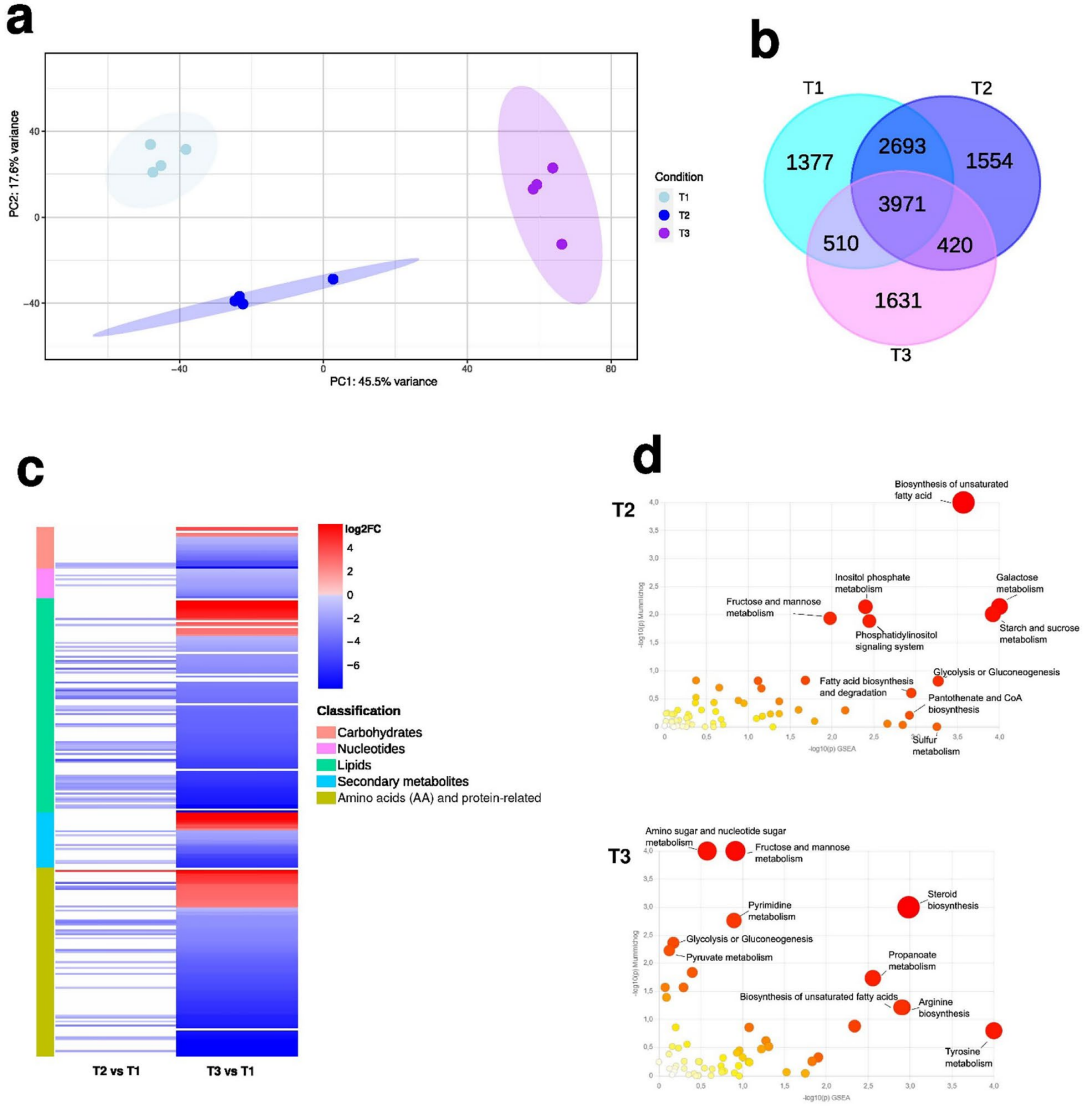
PCA, heatmap, and Venn diagrams depicting metabolites in the conditions T1, T2, T3, and functional analysis results. In **a**, PCA on detected metabolites in T1, T2, and T3. In **b**, the heatmap shows the expression patterns of metabolites in the two conditions T2 and T3 compared to T1, with red indicating up-regulated and blue representing down-regulated metabolites. Different color intensity indicates different relative amount based on Log_2_FC. In **c** Venn diagrams illustrate the overlap of detected mass features between pairwise comparisons of the conditions. In **d**, over-representation analysis shows enriched pathways (bubbles) in T2 and T3. Log_10_p-value of Mummichog and GSEA algorithms are represented by x and y axes, respectively

The metabolomic profiles of the mycelium at the pre-symbiotic stage T2 and at the symbiotic stage T3 were compared with the profile at the early stage T1. The mycelium metabolome showed a significant divergence between the early (T1), the pre- symbiotic (T2) and the symbiotic (T3) stages of growth, as shown by the segregation in the first two components of the PCA, which collectively explained 63% of the total metabolome variance (Fig. 5a). The metabolome of the SYMB sample was not considered because it contains both fungal and plant metabolites that cannot be distinguished, unlike in the gene expression analysis. In total, the metabolome analysis could detect 12,648 metabolite-related mass features among the different stages. Of the total number of metabolite-related mass features, 3,971 were shared between T1, T2 and T3 (Fig. 5b), and 2,508 additional mass features were differentially accumulated (VIP > 1; *p* < 0.05, CV-ANOVA; PLS-DA Fig. S1). A considerable number of mass features were shared between T1 and T2 (2,693), which may be indicative of metabolites specifically involved in the active growth phase of the mycelium. In contrast, the 420 mass features shared between T2 and T3 may be indicative of the influence of the plant on the fungal metabolome. Among the 2,508 differentially accumulated mass features, 88 could be matched by MS/MS data to chemical structures available in databases (Fig. 5c; Table S11). Furthermore, 281 mass features could be classified as ‘lipids’ (114, 40.6%), as ‘amino acids (AA) and protein-related’ (98, 35%), as ‘secondary metabolites’ (33, 12%), as ‘carbohydrates’ (20, 7%), or as ‘nucleotides’ (16, 6%) using the MSCC approach (Fig. 5c; Table S11). The heatmap (Fig. 5c) depicts the significant relative changes in the metabolome of T2 and T3 samples, in comparison to T1. Whereas minimal changes in the fungal metabolome were observed in T2, mainly a down-regulation, profound alterations were revealed in T3, with a specific and strong up-regulation (Log_2_FC > 4) of several metabolites involved in the metabolism of amino acid, lipids, and secondary compounds (Fig. 5c). Among the putatively annotated compounds that exhibited a significantly change (adj. *p* < 0.05; Table S11), several glycerophospholipids were identified in T3, including three glycerophosphocholines PC 36:3 (Log_2_FC: 7.34), PC 34:2 (Log_2_FC: 6.86) and PC 32:2 (Log_2_FC: 2.77). Among the secondary metabolites, the levels of indole-3-carboxylic acid (ICA; Log_2_FC: 4.88), the phenolic acid 3,4-dihydroxyphenylacetate (3,4-DHPA; Log_2_FC: 3.82), the alkyl-phenylketone 4-O-methylphloracetophenone (Log_2_FC: 3.77), and the pyranone 4-hydroxy-6-methyl-2-pyrone (Log_2_FC: 2.42) were significantly increased in T3. A slightly higher level of ICA was observed in T2, although this was not significant. Among the putatively annotated compounds that decreased significantly in both the pre-symbiotic T2 and the symbiotic T3 stages, in comparison to T1, were three lyso-phosphatidylcholines (LysoPC 14:0, LysoPC 18:3, LysoPC 16:0) and a phosphatidylcholine (PC 18:0). In addition, three glycerophosphoglycerols (LPG 18:2, LPG 16:0, LPG 18:1), a glycerophosphoinositol (PI 36:4), two glycerophosphates (PA 36:3 and LPA 16:0) and two medium-chain fatty acids (MCFAs), the 10-hydroxydecanoic acid and the sebacic acid, were also decreased. Other metabolites that exhibited reduced abundance during the later (T3) stages of fungal development included the modified amino acid 3-methyl-L-histidine (Log_2_FC T2: − 1.50 and Log_2_FC T3: − 4.59), which is known to be a structural component of actin and myosin (Webb et al. 2010), the amino acid arginine (Log_2_FC T2: − 2.2 and Log_2_FC T3: − 2.8) and sclareol, a labdane diterpenoid (Log_2_FC T2: − 1.75 and Log_2_FC T3: − 1.66). Finally, compounds exhibiting a slight decrease in T2 and a pronounced decrease in T3 included the oxidized form of glutathione (GSSG), the pyrimidine ribonucleoside diphosphate cytidine 5'-diphosphocholine (CDP), the tetra saccharide stachyose, and the fatty aldehyde succinic acid (Table S11). The remaining annotated compounds, which included several amino acids such as serine, lysine, histidine, arginine, isoleucine, taurine or phenylalanine, as well as carbohydrates such as xylose, gluconic acid or trehalose, showed significantly decreased levels in T3 in comparison to T1 (Table S11). According to our RNA-seq data (Table S9), a putative fungal trehalase (transcript ID: 20428) was significantly up-regulated in T2 (Log_2_FC = 1.68) and T3 (Log_2_FC = 1.62), suggesting a possible degradation of trehalose in the fungus.

### Changes in the metabolome suggest activation of energy, carbon and nitrogen metabolic pathways in the different stages

The over-representation analysis of significant metabolomic changes revealed that the fructose / mannose metabolisms and the biosynthesis of unsaturated fatty acids were significantly enriched in both stages T2 and T3 (combined *p* values < 0.05; Fig. 5d and Tables S12-S13). Nevertheless, some metabolic pathways were specific to each condition. At the pre-symbiotic stage T2, five pathways (combined *p* values < 0.05) were affected, the majority of these pathways being related to energy and carbon metabolism. This included galactose metabolism, starch and sucrose metabolism, inositol phosphate metabolism, phosphatidylinositol signalling, and glycolysis or gluconeogenesis (Fig. 5d and Table S12). In T3, six metabolic pathways were identified as being affected, mainly related to nitrogen metabolism. These pathways included steroid biosynthesis, amino sugar and nucleotide sugar metabolism, tyrosine metabolism, propanoate metabolism, arginine biosynthesis and pyrimidine metabolism (Fig. 5d and Tables S12-S13). When the results of the Mummichog 3.0 and the GSEA algorithms were considered separately, the T2 samples were found to be enriched in sulfur metabolism, fatty acid biosynthesis and degradation, and pantothenate and CoA biosynthesis (GSEA *p* values: 0.0339, 0.04651, 0.04651, 0.04762, respectively; Fig. 5d). In T3, glycolysis or gluconeogenesis, and pyruvate metabolism (Mummichog 3.0 *p* values: 0.03 and 0.04, respectively; Fig. 5d) were found to be enriched.

### Integration between transcriptomic and metabolomic data

**Fig. 6:**
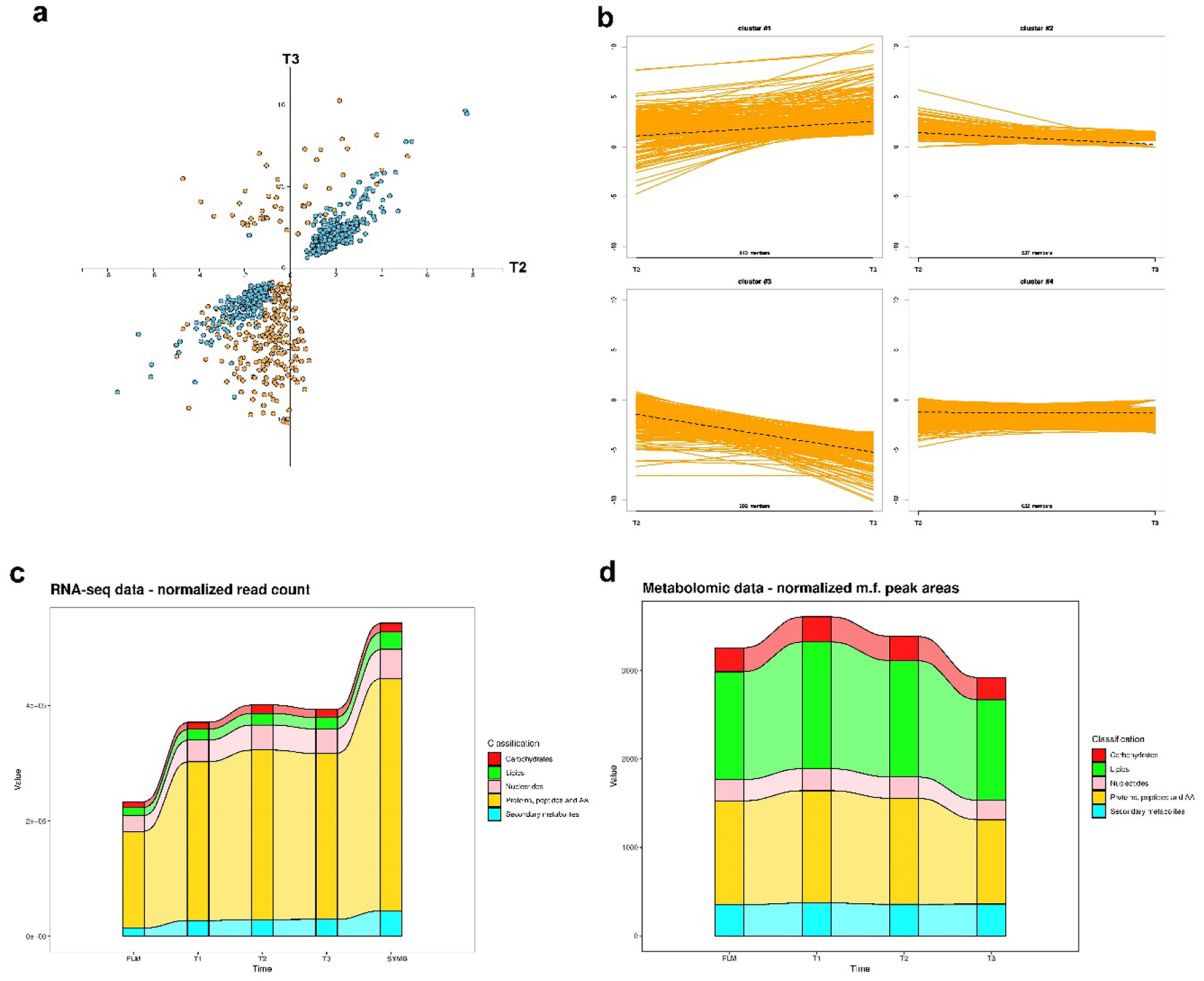
Integration of transcriptomic and metabolomic data. In **a**, scatterplot depicting the correlation of DEGs and metabolites (Log_2_FC) between T2 (x-axis) and T3 (y-axis). Blue dots represent DEGs, while yellow dots represent metabolites. In **b**, detected clusters through neural gas cluster analysis. In **c** and **d**, alluvial plots of normalized transcriptomic and metabolomic data across the different tested stages, respectively

A scatterplot of integrated transcriptomic and metabolomic data from T2 and T3 conditions demonstrated a strong correlation between gene expression and metabolomic data (R^2^ = 0.819, p-value = 6.06e^−12^; Pearson correlation). This indicates co-regulation of several genes and metabolites in the two conditions, particularly for down-regulated genes and metabolites (Fig. 6a). Notwithstanding the positive correlation, a group of metabolites showed down-regulation in T2 and up-regulation in T3 (Fig. 6a). Furthermore, neural gas cluster analysis facilitated the integration of transcriptomic and metabolomic datasets, resulting in the generation of 4 clusters based on the putative co-regulation of genes and metabolites (Fig. 6b). Among the identified clusters, cluster 3, which encompasses transcripts and metabolites showing a declining trend from T2 to T3 (Fig. 6b), is composed of a substantial number of mass features annotated as lipids, in addition to a DEG encoding a phospholipase A2. A list of metabolites and transcripts within the detected clusters is provided in Table S14. Alluvial plots of transcriptomic data (Fig. 6c) showed shifts in transcript abundance across the five categories over distinct time points. In particular, transcripts related to biosynthesis/catabolism of proteins, lipids and carbohydrates showed a consistent increase in abundance from FLM to T1, T2 and T3. A notable increase in secondary metabolite-related transcripts was found in SYMB, and low levels of transcripts related to this category was highlighted in FLM. On the other hand, concerning the metabolomic data, lipids and carbohydrates exhibited the highest levels in T1, while a decreasing trend was observed in T2 and T3 (Fig. 6d).

## Discussion

The application of *omics* techniques to investigate fungal-plant interactions has gained widespread recognition within the ORM community (De Rose et al. 2023b). In this study, we focused on the transcriptomic and metabolomic reprogramming of the ORM fungus *Tulasnella* sp. V6 during the early initial stages of its interaction with the host *Serapias vomeracea,* with the aim to elucidate the temporal molecular changes that occur in the fungus prior to and after contact with the plant. Thus, we have provided a wider picture of the ORM fungal behaviour in symbiosis, as previous investigations on ORM have only considered fungal gene expression and metabolite profiles in fully established symbiotic protocorms, as compared to FLM (Fochi et al. 2017a; Ghirardo et al. 2020; Chen et al. 2022).

Our integrated transcriptomic and metabolomic study indicates that the fungal mycelium initiates recognition of the host plant before physical contact, thereby preparing the fungal cells for establishment of the symbiosis. In particular, our results demonstrate the gradual activation of cell signalling pathways, the enhancement of the amino acid metabolism and nitrogen transport, as well as lipid remodelling and the expression of genes encoding plant cell wall-degrading enzymes (PCWDEs), which are responsible for the degradation of the constituents of the plant cell wall. It has been previously reported that nitrogen is transferred from *Tulasnella* sp. to *S. vomeracea* as amino acids (Fochi et al. 2017a, b). These processes are of great importance for the establishment and functioning of the symbiosis.

### *Tulasnella* sp. SV6 activates calcium-related signalling pathways during the pre-symbiotic stage

Genes involved in cell signalling pathways were expressed in *Tulasnella sp.* prior to the symbiotic stage. In T2, the up-regulation of two calmodulin-coding genes is noteworthy, particularly considering their role in intracellular calcium signalling (Yang and Poovaiah 2003). Calcium ions play a crucial role in cell signalling, specifically in the transduction of signals from the extracellular environment and in the activation of enzymes involved in gene expression (Roy et al. 2021). Calcium signalling response typically occurs at the initial stage of reaction to a stimulus, thus modulating fungal growth and development in response to the surrounding environment (Lengeler et al. 2000). In arbuscular mycorrhizal (AM) fungi, fungal genes related to calcium homeostasis and signalling are induced during symbiotic interactions with host roots, thus suggesting a role of Ca^2+^-based signalling pathways during the differentiation of the hyphopodium, a specialized specialized hypha of an arbuscular mycorrhizal fungus, often branched and swollen, which adheres to the root epidermis (Bonfante and Genre, 2010). In our data, up-regulation of genes coding for calmodulins and several kinase proteins in T2 would indicate that the fungus perceives the presence of the orchid seeds and activates an intracellular Ca^2+^-signalling pathway prior to contact, possibly through recognition of chemical signals released from the imbibed seeds (Ortega-Cuadro et al. 2022). However, the actual existence and chemical structure of these plant signals remain unknown and warrant further dedicated studies.

Among the most up-regulated genes involved in signal transduction in T2, a putative Ras guanine-nucleotide exchange protein was also identified. This protein is responsible for the activation of Ras proteins by promoting the binding of GTP to the GDP-free Ras forms (Chen et al. 2020). The Ras superfamily, which includes monomeric GTPases, regulates a multitude of biological processes in fungi, including morphogenesis, secondary metabolism, vesicle trafficking and virulence (Dautt-Castro et al. 2021; Pinar and Peñalva 2021; Yuan et al. 2022). The most common members of the Ras superfamily are Ras, Rho, Rab, Ran, Arf, and Miro (Cao et al. 2024; Xu et al. 2024). The activity of these GTPases within the cell is regulated by guanine-nucleotide exchange factors (GEFs) and by GTPase activating proteins (GAPs), which respectively catalyze binding of GTPase with either GTP (active form) or GDP (inactive form). The Ras signalling pathway, in particular, is responsible for orchestrating a range of cellular processes that are essential for hyphal growth in fungi (Martin-Vicente et al. 2019; Chen et al. 2020).

Intriguingly, Breuninger and Requena (2004) observed the up-regulation of genes involved in Ca^2+^ signalling and in the Ras signalling pathway in the fungal partner of AM symbioses during the early stages of appressorium development. Arbuscular mycorrhiza is the most common endomycorrhizal type (Genre et al. 2020), and notable similarities with ORM have been outlined, despite the phylogenetic distance of the plants and fungi involved. For example, orchids feature in their genome genes of the Common Symbiotic Signalling Pathway, a conserved signal transduction pathway found in all endosymbioses (Delaux et al. 2014; Miura et al. 2018). Furthermore, similarities among the intracellular structures formed by AM and ORM fungi in their respective hosts have been recently discussed (Perotto and Balestrini 2024). In AM, two main types of root colonization patterns were generally observed, the *Arum*-type and the *Paris*-type. Whereas the *Arum*-type AM are characterized by highly branched intracellular fungal structures called arbuscules, intracellular fungal structures formed in *Paris*-type AM are simpler and more similar to ORM pelotons. In addition, intracellular fungal structures formed in both AM and ORM show a similar life cycle, including a collapse phase (Perotto et al. 2014).

Beside physical contact, chemical signalling between the plant and the fungus likely plays a role in activating signal transduction pathways. In AM, it has been documented that plants exude strigolactones, which are sensed by AM fungi and promote spore germination, hyphal branching, and other biological processes (Genre et al. 2020). During the establishiment of the symbiosis, AM fungi release chitin-derived molecules, called Myc factors, that trigger plant responses preparing them for colonization, including the fine tuning of expression of symbiosis-supporting genes as well as changes at cellular and physiological levels (Genre et al. 2020). Although presymbiotic chemical signalling is well studied in AM, in ORM the specific signalling molecules exchanged between host plants and their fungal partners are still largely unidentified (Genre et al. 2020; Perotto et al. 2014). The activation of a similar signal transduction pathways between AM and ORM is intriguing, and further analyses are needed to elucidate this poorly explored aspect. In our work, it is noteworthy that metabolomic data indicate two unique enriched pathways at the pre-symbiotic stage (T2), namely inositol phosphate metabolism and the phosphatidylinositol signalling system. These pathways are putatively involved in signal transduction and cellular communication and may also be indicative of the activation of signalling processes during the pre-symbiotic stage.

Mycorrhizal fungi, including ORM fungi, feature in their genomes a large set of mycorrhiza-induced small secreted proteins (MiSSPs), which can be highly expressed in symbiosis (Kohler et al. 2015; Kang et al. 2020; Miyauchi et al. 2020). Some MiSSPs have been characterized in AM, ecto- and ericoid mycorrhizal fungi and have been demonstrated to be involved in the establishment of the symbiosis and/or in the inactivation of the plant defense mechanisms (Kloppholz et al. 2011; Plett et al. 2011; Casarrubia et al. 2018). In *T. calospora*, several genes strongly upregulated during the symbiosis with orchids encoded putative MiSSPs (Kohler et al. 2015). Here, we identified two DEGs up-regulated in the mycelium in the proximity of symbiotic protocorms (T3), one of which (transcript ID 242048) was also up-regulated in pre-symbiotic condition (T2) but down-regulated in the protocorms (SYMB). The relatively low number of up-regulated genes coding for MiSSP in our model system may be attributed to the fact that MiSSP tend to be highly species-specific (Pellegrin et al. 2015; Kim et al. 2016). Thus, we may have missed the annotation of species-specific MiSSP-related genes when we compared the *Tulasnella* sp. isolate SV6 (used in this study) with the genome of the sequenced *T. calospora* isolate available in Mycocosm. Anyhow, the two genes identified in this study warrant further investigation, as they may represent good candidates for the study of symbiosis effectors in ORM fungi.

### Response to the host plant involves increase protein synthesis and transport in the pre-symbiotic stage

Among the fungal genes regulated prior to physical contact with the orchid seeds, we identified genes involved in amino acid metabolism, including an asparagine and a methionine synthase. Together with the specific enrichment of the GO term “Translation” in T2 and the up-regulation of several genes involved in ribosome biosynthesis, this suggests an increased primary metabolic activity in the pre-symbiotic stage, possibly associated with changes in fungal development. Not surprisingly, given the intense metabolic changes triggered by the ORM symbiosis, sustained translation activity continued in the fungal mycelium after physical contact both outside and inside mycorrhizal protocorms.

Some fungal genes coding for membrane transporters or related to transport were also significantly up-regulated in T2, before physical contact with the host. In particular, 19 DEGs related to transporters were identified in the pre-symbiotic stage, suggesting transport activity for amino acids and oligopeptides. Membrane transporters in fungi are essential for the uptake of nutrients from the substrate and their subsequent redistribution within cellular compartments. In mycorrhizal fungi, they also play a pivotal role in nutrient exchanges with the host during symbiosis (Garcia et al. 2016; Wipf et al. 2019), and several transporters were identified in symbiosis in this and in previous studies (Zhao et al. 2014; Fochi et al. 2017a; De Rose et al. 2023a, b). Although it cannot be ruled out that, in the pre-symbiotic stage T2, up-regulation of fungal genes involved in translation and transport may simply reflect changes in substrate composition during fungal growth, it should be noted that expression of these genes was similar in T1, where the mycelium just started to colonize the substrate, and in FLM, where the fully grown mycelium covered the whole plate. Thus, if modulation of these fungal activities in the pre-symbiotic condition is related to changes in fungal growth, these changes appear to be caused by the proximity of orchid seeds. Of course, this hypothesis would suggest the release of so far unknown diffusible signals from the seeds.

### Several CAZymes and PCDWEs are expressed in pre-symbiotic stages

CAZymes, and plant cell wall-degrading enzymes (PCWDEs) in particular, are instrumental for the degradation of the polymeric litter components by saprotrophic fungi. However, they also play a significant role during symbiosis (Kohler et al. 2015; Martin et al. 2016; Almario et al. 2017; Genre et al. 2020). A distinctive feature of ORM fungi such as *Tulasnella calospora* is their larger set of PCWDEs (Kohler et al. 2015) in comparison to other mycorrhizal fungi. It has been proposed that, thanks to these degrading enzymes, ORM fungi may maintain a double ecological niche, with saprotrophic and symbiotic lifestyles (Martino et al. 2018; Miyauchi et al. 2020) In addition, ORM fungi may also live as endophytes in the roots of non-orchid plants (Selosse et al. 2022).

In our experiment, comparison of gene expression with T1 samples (i.e., the early *Tulasnella* growth stage on fresh medium in the presence of orchid seeds) reduced the possibility that the DEGs identified were simply related to the exploitation of carbohydrates available in the substrate. In fact, whereas only one DEG corresponding to a Glycoside Hydrolase (GH5) was identified in FLM when compared to T1, several CAZyme-related genes were up-regulated in the pre-symbiotic stage (T2) and in the external mycelium in the proximity of symbiotic protocorms (T3). In comparison to T1, 14.7% and 21% of the total number of CAZymes found in the *T. calospora* AL-13/4D v1.0 genome were induced in T2 and T3, respectively. The percentage of up-regulated CAZymes related fungal genes was considerably higher (47% of the total number of CAZymes in the T. calospora AL-13/4D v1.0 genome) in symbiotic protocorms (SYMB).

The up-regulation of a chitin synthase (GT2, transcript ID: 79704) unique to the pre-symbiotic stage is of particular interest, as it suggests an active remodelling of the fungal cell wall, possibly related to changes in the growth of fungal mycelium upon perception of the orchid seeds. It is noteworthy, that chitin synthase is also involved in the synthesis of lipochitooligosaccharides and short chitin oligomers, which are molecular signals released by AM fungi in the pre-symbiotic stages of the interactions (Maillet et al. 2010; Genre and Russo 2016). In addition, other genes related to glycoside hydrolases and glycosyl transferases and possibly involved in fungal cell wall remodelling (Bowman and Free 2006) were up-regulated in the pre-symbiotic stage.

The role of fungal CAZymes in the ORM symbiosis remains unclear. Several glycosyl hydrolases in the GH5 family and polysaccharide lyases in the PL1 family were induced at an early stage before symbiosis, and their number increased as the fungus established ORM symbiosis (T3 and SYMB). A set of enzymes belonging to the GH5 family was identified in the genome of the ectomycorrhizal fungus *Laccaria bicolor*, and were shown to be active on both fungal and plant cell walls (Veneault-Fourrey et al. 2014). Enzymes in the PL1 family include pectin and pectate lyases and are known to play a crucial role in plant cell wall degradation (Atasanova et al. 2018). Our RNAseq data thus suggest that GH5 and PL1 enzymes may be involved in modifying the orchid cell wall, potentially facilitating the symbiotic interaction. In SYMB condition, within the 83 CAZymes-related genes resulted as DEGs, 30 PCWDE-related genes were identified as up-regulated, suggesting that they might be involved in the fungal colonization and establishment of the symbiotic interface. This outcome also suggests that the plant cell wall degradative potential of *Tulasnella* is finely tuned during symbiosis, as previously reported (Adamo et al. 2020).

### Remodelling of the fungal lipidic profiles during the pre-symbiotic and symbiotic stages

The enrichment analysis of metabolomic data revealed that biosynthesis of unsaturated fatty acids was induced in the *Tulasnella* mycelium before the fungus came in physical contact with the seeds (T2), and persisted when symbiosis was established (T3). Increased abundance of structural membrane lipids may reflect the stimulation of hyphal growth and a need for membrane biogenesis in the mycelium during both the T2 and T3 stages. However, the increase in potential membrane signalling molecules is intriguing. Phospholipids such as phosphatidylcholines (PC) are the dominant membrane lipids in fungi (Rella et al. 2016). In plants, a derivative of phosphatidylcholine, lyso-phosphatidylcholine (Lyso-PC) is known to act as a signal in the AM symbiosis (Drissner et al. 2007; Vijayakumar et al. 2015). In our experimental system, the amounts of metabolites putatively related to Lyso-PCs (Lyso-PC 14:0, Lyso-PC 18:3, Lyso-PC 16:0) were found to be higher in the early stages of co-cultivation, as compared to both FLM and T3. Lyso-PC content particularly decreased in T3, and was mirrored by an increase in the amount of PC. Intriguingly, the transcriptomic-metabolomic cluster analysis revealed that the decline of Lyso-PC levels in T3 correlated with the down-regulation of a gene encoding a phospholipase A2 (with Log_2_FC of  − 4.73). Cleavage of PC by phospholipase A2 enzyme is the main process for the generation of Lyso-PC (Liu et al. 2020). Overall, these results indicate that Lyso-PCs may be involved during the pre-symbiotic stages of the *Tulasnella*-*Serapias* symbiosis, similar to what has been observed in the AM symbiosis (Vijayakumar et al. 2015). Once the symbiosis is established, Lyso-PCs appear to be depleted in the mycelium collected in close proximity to symbiotic protocorms (T3). Ghirardo et al. (2020) reported a significant up-regulation of the fungal phospholipase A2 gene in symbiotic protocorms, in comparison to FLM, but the substrate of this enzyme is unknown.

### An indole related metabolite is produced before fungal contact with orchid seeds

In comparison to the very early T1 stage, stages T2 and T3 of the *Tulasnella*-*Serapias* interaction were characterised by a substantial increase (Log_2_FC of 1.70 and 4.88, respectively) of a compound putatively annotated as indole-3-carboxylic acid (ICA). Indole-related compounds released from mycorrhizal fungi have been reported to regulate establishment of the symbiosis through lateral root formation (Ludwig-Müller 2010; Hanlon and Coenen 2011; Sukumar et al. 2013; Liao et al. 2018). Among indole compounds, ICA can be produced by ectomycorrhizal fungi, including *Paxillus involutus* and *Suillus luteus* (Rudawska and Kieliszewska-Rokicka 1997; Pan et al. 2022), and may promote mycorrhizal symbiosis (Wu et al. 2021). To the best of our knowledge, our data represents the first report of ICA in the genus *Tulasnella*, and its increased production prior to and during the establishment of the ORM symbiosis suggests similarities to its role in ectomycorrhizal fungi (Wu et al. 2021). However, manipulation of the endogenous levels of fungal ICA would be essential to explore its role in the ORM symbiosis. This could be achieved in future studies through the application of exogeneous ICA, of specific enzyme inhibitors, or by genetic manipulation of its biosynthesis.

## Conclusions

Our integration of transcriptomic and metabolomic data revealed the molecular changes that occur in *Tulasnella* sp. SV6 before and after physical contact with the orchid host *S. vomeracea*. In particular, the significant up-regulation of genes and metabolites potentially involved in early signalling suggests that the ORM fungus senses and responds to the presence of the host before physical contact with the orchid seeds. Early perception of orchid seeds by the fungus was further suggested by the increased production of specific fungal compounds such as an indole-related metabolite, putatively involved in the early stimulation of symbiosis.

Transcriptomics and metabolomics showed the up-regulation of some primary and secondary metabolic pathways during the initial stages of the symbiotic interaction. These pathways likely play a role in modulating fungal growth in response to the presence of orchid seeds. Furthermore, the up-regulation of *Tulasnella* genes related to PCWDEs during early contact with the *Serapias vomeracea* seeds supported the hypothesis that these enzymes play a role in fungal penetration and host cell colonization. Overall, our integrated approach enabled the identification of the dynamic nature of the ORM symbiosis, and the generation of useful data on the fungal side, which remains a relatively understudied topic (Favre-Godal et al. 2020).

## Supplementary Information

Additional file 1.

Additional file 2.

## Data Availability

Transcriptomic raw data were submitted to Sequence Read Archive (SRA) of NCBI, under the BioProject ID: PRJNA979980.

## Abbreviations

AM fungi: Arbuscular mycorrhizal fungi
CAZymes: Carbohydrate-active enzymes
DEGs: Differentially expressed genes
GO: Gene ontology
GH: Glycoside hydrolase
GSEA: Gene set enrichment analysis
HILIC: Hydrophilic interaction liquid chromatography
ICA: Indole-3-carboxylic acid
KEGG: Kyoto encyclopedia of genes and genomes
KOG: EuKaryotic orthologous groups
Lyso-PC: Lyso-phosphatidylcholine
MUT: Mycotheca Universitatis Taurinensis
MSCC: Multidimensional stoichiometric compound classification
ORM fungi: Orchid mycorrhizal fungi
PCA: Principal component analysis
PCWDE: Plant cell wall-degrading enzyme
PL: Polysaccharide lyase
PLS-DA: Partial least squares discriminant analysis
RPLC: Reversed-phase liquid chromatography
VIP: Variable importance of projection
