# Supplementary figures and images for: Integration of fungal transcriptomics and metabolomics provides insights into the early interaction between the ORM fungus Tulasnella sp. and the orchid Serapias vomeracea seeds
Source: IMA Fungus. 2024 Oct 25;15:31. doi: 10.1186/s43008-024-00165-6 (PMC11503967; doi:10.1186/s43008-024-00165-6)

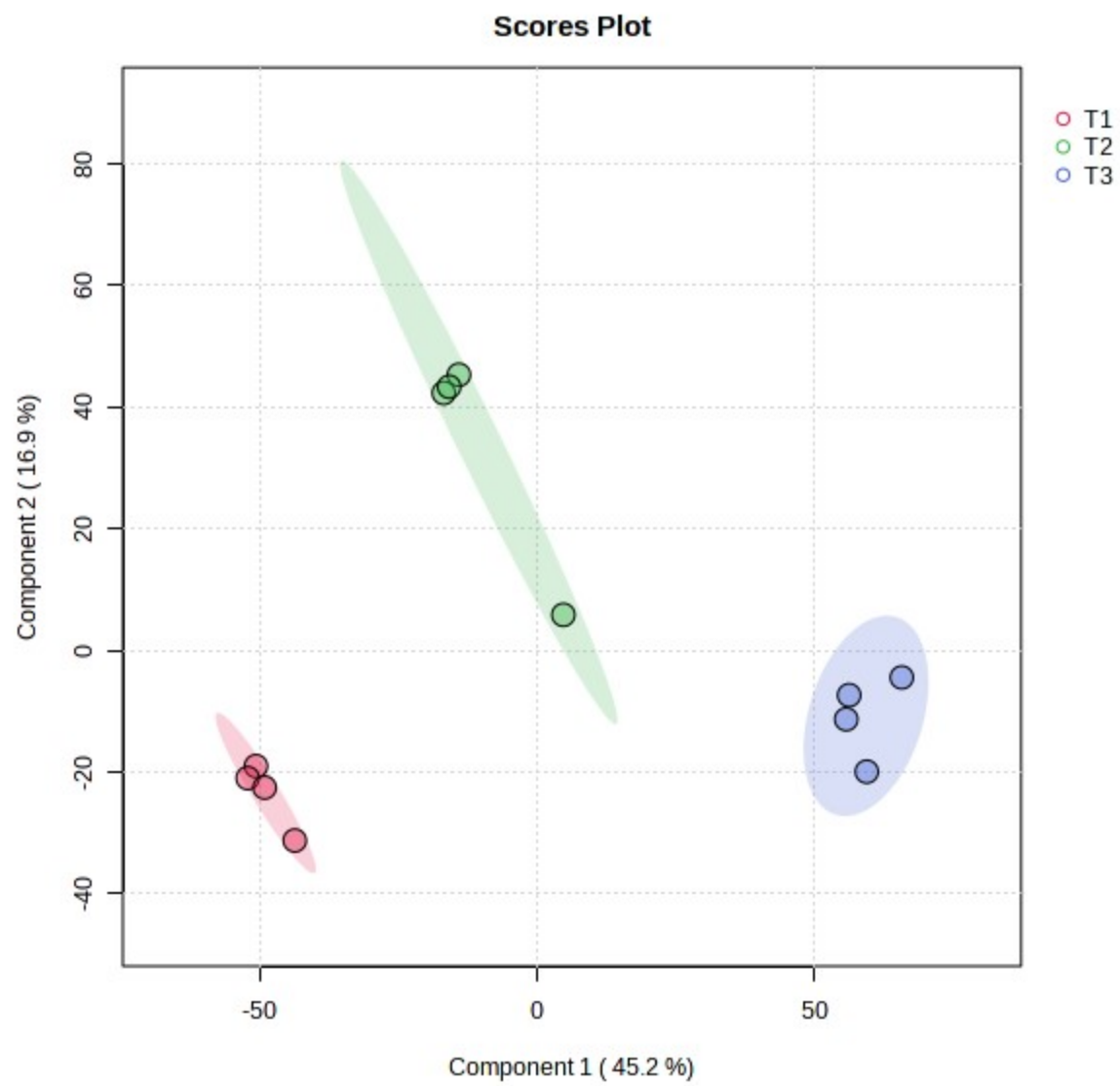

Supplement: Supplementary file 1 — Additional file 1. [file 43008_2024_165_MOESM1_ESM.pdf]
